# AI-driven pan-proteome analyses reveal insights into the biohydrometallurgical properties of *Acidithiobacillia*

**DOI:** 10.3389/fmicb.2023.1243987

**Published:** 2023-09-07

**Authors:** Liangzhi Li, Lei Zhou, Chengying Jiang, Zhenghua Liu, Delong Meng, Feng Luo, Qiang He, Huaqun Yin

**Affiliations:** ^1^School of Minerals Processing and Bioengineering, Central South University, Changsha, China; ^2^Key Laboratory of Biometallurgy of Ministry of Education, Central South University, Changsha, China; ^3^Beijing Research Institute of Chemical Engineering and Metallurgy, Beijing, China; ^4^State Key Laboratory of Microbial Resources, Institute of Microbiology, Chinese Academy of Sciences, Beijing, China; ^5^University of Chinese Academy of Sciences, Beijing, China; ^6^School of Computing, Clemson University, Clemson, SC, United States; ^7^Department of Civil and Environmental Engineering, University of Tennessee, Knoxville, Knoxville, TN, United States

**Keywords:** proteome, biohydrometallurgy, protein structure prediction, gene co-occurrence network analysis, *Acidithiobacillia*

## Abstract

Microorganism-mediated biohydrometallurgy, a sustainable approach for metal recovery from ores, relies on the metabolic activity of acidophilic bacteria. *Acidithiobacillia* with sulfur/iron-oxidizing capacities are extensively studied and applied in biohydrometallurgy-related processes. However, only 14 distinct proteins from *Acidithiobacillia* have experimentally determined structures currently available. This significantly hampers in-depth investigations of *Acidithiobacillia*’s structure-based biological mechanisms pertaining to its relevant biohydrometallurgical processes. To address this issue, we employed a state-of-the-art artificial intelligence (AI)-driven approach, with a median model confidence of 0.80, to perform high-quality full-chain structure predictions on the pan-proteome (10,458 proteins) of the type strain *Acidithiobacillia*. Additionally, we conducted various case studies on *de novo* protein structural prediction, including sulfate transporter and iron oxidase, to demonstrate how accurate structure predictions and gene co-occurrence networks can contribute to the development of mechanistic insights and hypotheses regarding sulfur and iron utilization proteins. Furthermore, for the unannotated proteins that constitute 35.8% of the *Acidithiobacillia* proteome, we employed the deep-learning algorithm DeepFRI to make structure-based functional predictions. As a result, we successfully obtained gene ontology (GO) terms for 93.6% of these previously unknown proteins. This study has a significant impact on improving protein structure and function predictions, as well as developing state-of-the-art techniques for high-throughput analysis of large proteomic data.

## 1. Introduction

Biohydrometallurgy, including bioleaching and biomining, involves the accelerated dissolution of sulfidic minerals by acidophilic chemolithotrophic microorganisms to recover metals. In biohydrometallurgy operations, pulverized copper ores are piled up, inoculated with solutions containing specific microbiota and sulfuric acid, and aerated to facilitate the microbial oxidation of iron and sulfur compounds (Valdés et al., 2008). *Acidithiobacillia*, a member of the earliest and most extensively studied microbial consortia, has been widely employed in various biohydrometallurgy processes (Banderas and Guiliani, 2013; Campodonico et al., 2016; Li et al., 2019; Inaba et al., 2020). *Acidithiobacillus* is the type genus of the order *Acidithiobacillales* (type order of the class *Acidithiobacillia*) (Parte, 2014). *Acidithiobacillus* displays the central traits (e.g., sulfur/iron oxidation, CO_2_ fixation, heavy metal resistance) of the deep-branching *Proteobacteria* class *Acidithiobacillia* (Moya-Beltrán et al., 2021). The representative species of *Acidithiobacillus*, *Acidithiobacillus ferrooxidans*, is a Gram-negative, strictly acidophilic, chemolithoautotrophic bacterium that thrives optimally at temperatures around 30°C and pH levels of 1.8–2.2. It is commonly found in acidic environments such as acidified mineral drainages, coal deposits, and sulfuric springs (Moya-Beltrán et al., 2021). In our previous studies, we found that frequent horizontal gene transfer (HGT) of genes vital for survival, such as heavy metal resistance, have driven the adaptation of *Acidithiobacillia* to hostile biohydrometallurgy environments (Li et al., 2019; Zhang et al., 2019). *Acidithiobacillia* exhibit remarkable abilities in the efficient dissimilatory oxidation of various reduced inorganic sulfur compounds (RISCs) (Wang et al., 2019b) and are resistant to heavy metals (Li et al., 2019). Additionally, *Acidithiobacillia* can grow by oxidizing ferrous iron Fe(II) to ferric iron Fe(III) in acidic solutions, with oxygen serving as the terminal electron acceptor (Liu et al., 2013). These combined physiological traits (sulfur and iron oxidation, acid and metal resistance) account for the widespread commercial application of *Acidithiobacillia* in biotechnologies related to the dissolution of sulfide and metallic minerals, as well as the extraction of valuable metals (Zhang et al., 2018a). Furthermore, researchers are interested in modifying *Acidithiobacillus* to become an electrochemically active bacterium (EAB) for recycling electronic waste (Wang et al., 2009) and for biofuel production from carbon dioxide using reduced iron as the sole energy source (Guan et al., 2017).

The various characteristic abilities and other life-sustaining aspects of *Acidithiobacillia* are determined by the protein machinery it encodes and expresses (Ramírez et al., 2004; Vera et al., 2013). The sulfur oxidation pathway of *Acidithiobacillia* typically involves several steps: sulfide species are oxidized to elemental sulfur by sulfide:quinone oxidoreductase (SQR), sulfide species can be converted to sulfite through sulfite reductase (Dsr), tetrathionate is converted to sulfite via tetrathionate hydrolase (Ttr) and sulfotransferase, sulfite is reversibly oxidized to sulfate through adenylylsulfate reductase (AprA) and sulfate adenylate transferase (SAT), and sulfate is transported using a sulfate transporter. Additionally, *Acidithiobacillia* primarily relies on proteins encoded by the rus gene operon (with rusticyanin as the core protein) and a high potential iron-sulfur protein (HiPIP), encoded by Iro, for iron oxidation. The HiPIP protein acts as the primary electron acceptor from Fe(II) in an alternative electron transfer pathway (Bruscella et al., 2005; Valdés et al., 2008; Quatrini et al., 2009). Over the last few decades, researchers have resolved the structure of 14 different proteins from *Acidithiobacillia* [search of the Protein Data Bank (PDB) database (Goodsell et al., 2020) with the query keyword “*Acidithiobacillus/Acidithiobacillia*”], the majority of which are involved in metabolisms of energy substrates (e.g., sulfur compounds and iron). For instance, Botuyan et al. (1996) and Walter et al. (1996) characterized the structure of the iron oxidation protein rusticyanin from *A. ferrooxidans*, which provided insights into the mechanism of its enhanced acid stability and redox potential. This was soon followed by the structure of electron transfer protein C(4)-Cytochrome of *A. ferrooxidans*, resolved by Abergel et al. (2003) and then, Cherney et al. (2010, 2012) determined the structure of sulfide:quinone oxidoreductase and its variants from *A. ferrooxidans*, from which a novel reaction mechanism utilizing the Cys-S-S as the nucleophile to attack the cofactor was proposed. More recently, the crystal structure of tetrathionate hydrolase from *A. ferrooxidans* was resolved, which suggested a novel cysteine-independent tetrathionate hydrolysis mechanism (Kanao et al., 2021). Despite these efforts, the majority of other proteins from *A. ferrooxidans* still lack three-dimensional (3D) structures. This includes proteins directly involved in sulfur/iron utilization, such as the sulfate transporter and ferrous iron transporter, which play a crucial role in its biohydrometallurgy ability. This lack of protein structure data hinders further investigations into the molecular mechanisms of these proteins. One likely reason for this is that the experimental determination of a protein’s structure remains a time-consuming and expensive process (Bill et al., 2011; Lin, 2018).

Two influential artificial intelligence (AI)-driven algorithms, AlphaFold2 (Senior et al., 2020) and RoseTTAFold (Baek et al., 2021), have demonstrated their abilities to crack the long-lasting “protein-folding challenge.” Both show the strength to predict a wide range of complicated protein structures accurately and quickly using solely the amino acid sequences. *Homo sapiens* was the first species whose proteome to be extended to a structural coverage scale that encompasses its near entirety (98.5%) by employing the above-mentioned AI-based algorithm predictions (Tunyasuvunakool et al., 2021). However, myriads of other organisms including industrially important and biologically significant species like *A*. *ferrooxidans* are still highly underrepresented in the PDB database. Researchers of these organisms would be greatly benefited if the structures of their proteome are made available. Against this background, we choose the *Acidithiobacillia* pan-proteome (Parte, 2014) as our research subject, and expanded the structural coverage of the *Acidithiobacillia* to the entire pan-proteome (10,458 proteins) with full-chain predictions through application of the advanced AI-driven program AlphaFold2 (Senior et al., 2020) and RosettaFold (Baek et al., 2021).

The objective of this study was to predict the structure of the complete proteome (10,458 proteins) of *Acidithiobacillia*. The study also aimed to conduct case studies on the sulfur/iron utilizing processes, which are currently not well understood, and to provide raw structural data that can be further analyzed in detail. These investigations have significant scientific implications for enhancing predictions of protein structure and function, and for advancing advanced techniques for analyzing large proteomic datasets.

## 2. Results

### 2.1. Protein clustering and full-length protein structure predictions

**FIGURE 1:**
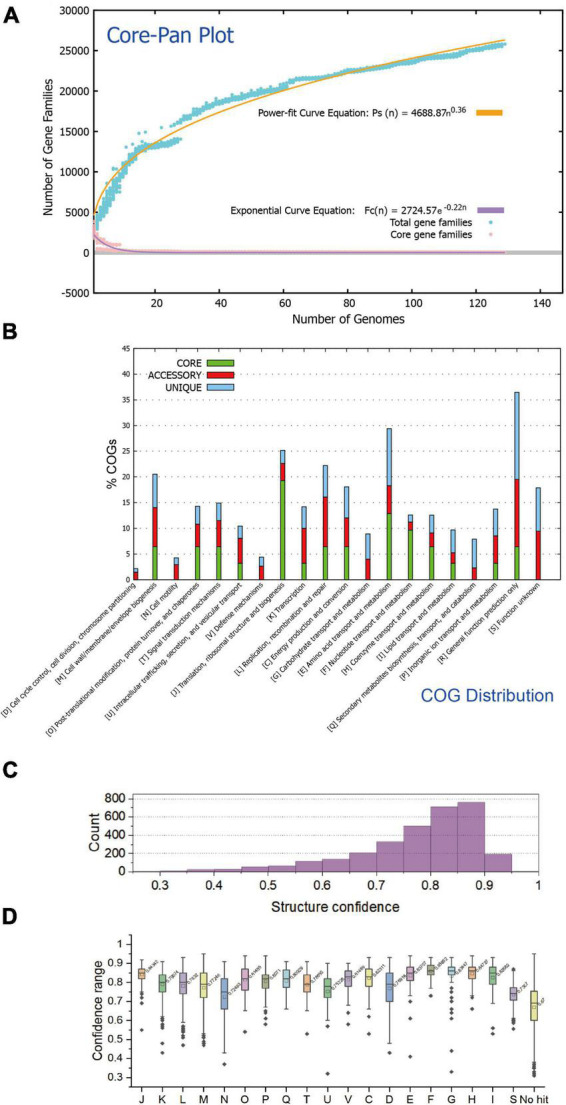
The statistical analysis of protein traits and structure confidences of the *Acidithiobacillia* proteome. **(A)** Mathematical modeling of the pangenome and core genome of *Acidithiobacillia*. **(B)** Bar chart showing functional proportions (based on COG categories) of different parts of the *Acidithiobacillia* pangenome (i.e., core, accessory, and unique). **(C)** Histogram showing the distribution of structure confidences of *Acidithiobacillia* pan-proteome. **(D)** Box plot showing the distribution of structure confidences of *Acidithiobacillia* pan-proteome among different COG categories with the average confidence values indicated.

A total of 129 available genomes (isolate) of the class *Acidithiobacillia* were obtained for protein clustering and pangenome analysis. The pangenome of the 129 *Acidithiobacillia* genomes consisted of 10,458 gene families, while the core genome contained 29 gene families (Figure 1A). Analysis of the core and pangenome revealed that the pangenome followed a power-law regression function [*Ps* (n) = 4688.87 n^0^.^36^], indicating an “open” pangenome. On the other hand, the core genome followed an exponential regression [*Fc* (n) = 2724.57 e^–0^.^22n^] (Figure 1A). The open pangenome suggests that *Acidithiobacillia* species may undergo gene exchange in order to enhance their functional profiles. The functional COG annotation (Figure 1B) reveals that the core genome has a higher proportion of genes classified in COG categories J (translation, ribosomal structure, and biogenesis), C (energy production and conversion), O (posttranslational modification, protein turnover, and chaperones), F (nucleotide transport and metabolism), and H (coenzyme transport and metabolism), which are associated with fundamental biological functions. On the other hand, the accessory genome and strain-specific genes are skewed toward COG categories G (carbohydrate transport and metabolism), L (replication, recombination, and repair), P (inorganic ion transport and metabolism), and N (cell motility). It is likely that these categories are linked to the adaptation of *Acidithiobacillia* to oligotrophic, metal-laden, and acidic environments, which can cause DNA damage.

We predicted structures for the pan-proteome of *Acidithiobacillia*. The resulting structural dataset covers the whole pan-proteome (10,458 proteins) with full-chain predictions. The predictions made by AlphaFold2 (Senior et al., 2020) agreed well with those made by RoseTTAFold (Baek et al., 2021), indicating reliable predictions. The comparison results show that the predicted models from both methods agreed well with each other, giving average pairwise TM-score of 0.93 and average pairwise root mean square deviations (RMSD) of 1.58. The average and median of model confidences are 0.77 and 0.80, respectively, with 69.4% (2,183/3,147) of all predicted models having a confidence over 0.75, and among these models, 44.4% (969/2,183) have a confidence over 0.85 (Figure 1C). The predicted model of ACK80295 (GNAT family acetyltransferase) had the highest confidence 0.95 (Supplementary Figure 1). Regarding COG categories, proteins assigned to COG F (nucleotide transport and metabolism), COG H (coenzyme transport and metabolism), and COG J (translation, ribosomal structure, and biogenesis) had the highest average confidences (0.86, 0.85, and 0.84, respectively) (Figure 1D). Additionally, we found that the prediction confidence was not correlated with protein sequence length (data not shown).

### 2.2. Highlight of predicted structures

**FIGURE 2:**
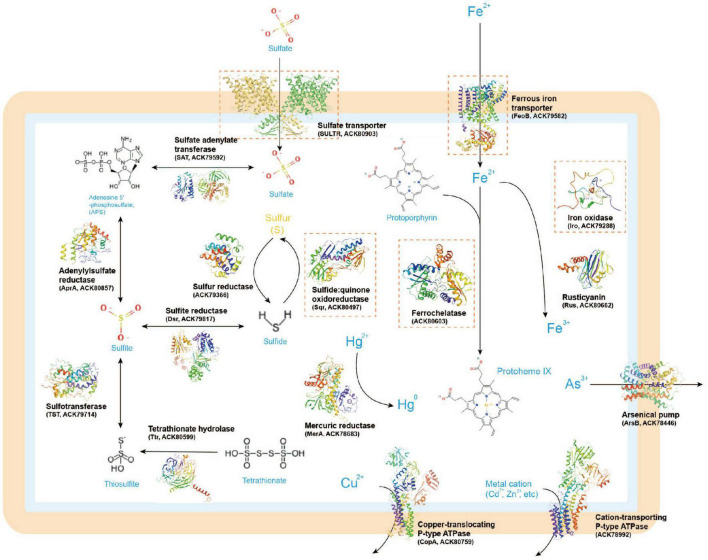
A metabolic model of sulfur/iron utilization/biometallurgy related proteins in *Acidithiobacillia* based on genomic inference. The 3D protein structures illustrated in the figure were predicted by AlphaFold2. The case-study proteins discussed in this study are marked with orange rectangles.

Next, we present and discuss several case-study predictions that focus on unresolved sulfur and iron transport and utilization proteins in *Acidithiobacillia*. These predictions may offer novel insights into the molecular mechanisms of this organism related to biohydrometallurgy. In the Methods section, we provide a summary of the detailed methods employed for these analyses, including substrate binding and molecular dynamics (MD) simulations. It is important to note that the predictions presented here are mainly *de novo*, meaning that no template with more than 30% query identity or covering over 35% of the sequence was available. These predictions can help bridge the knowledge gaps in our understanding of the functional roles and molecular details of these sulfur/iron utilization proteins within the broader biometallurgy system (see Figure 2, with the case-study proteins highlighted in orange rectangles). Although our results have a significant impact on improving protein structure and function predictions, as well as developing state-of-the-art techniques for high-throughput analysis of large proteomic data, experimental confirmation is ultimately necessary to determine the actual functions of the structure models and the hypothetical key residues within them.

#### 2.2.1. Sulfate transporter

**FIGURE 3:**
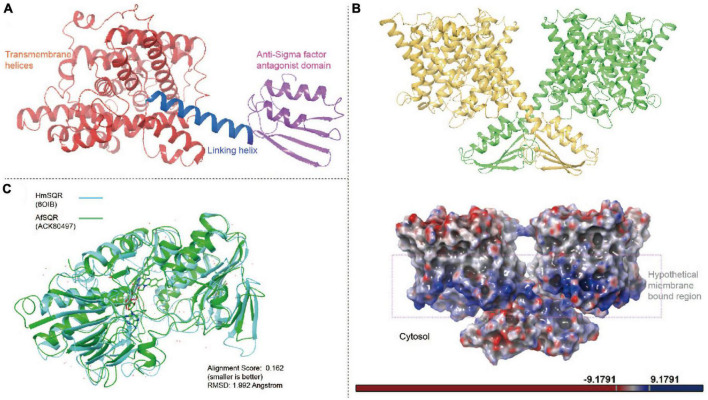
The structures of *Acidithiobacillia* sulfate transporter (AfSULTR-ACK80903) and sulfide:quinone oxidoreductase (AfSQR-ACK80497). **(A)** The overall structure of AfSULTR monomer is comprised of transmembrane (TM) helices and anti-Sigma factor antagonist (STAS) domain (shown with red and purple colors, respectively). **(B)** Top panel: the AfSULTR dimer model formed by two identical monomers (shown with yellow and green colors, respectively). Bottom panel: electrostatic potential surfaces of the overall AfSULTR dimer calculated with adaptive Poisson-Boltzmann solver (APBS). **(C)** Superposition of AfSQR-ACK80497 onto human SQR (HmSQR, PDB: 6OIB).

Sulfate (SO_4_^2–^), generated by the microbial oxidation of sulfide minerals, is the dominant aqueous sulfur species in the biomining drainage system and it is vital for microbes to maintain cellular sulfate homeostasis (Borilova et al., 2018). The sulfate transporter is identified in 81.5% of tested *Acidithiobacillia* genomes and has genomic location highly conserved among *Acidithiobacillia* (Supplementary Figure 2). The representative protein of it under Genbank (Benson et al., 2018) accession ACK80903 shows 25.32% sequence identity (the best hit in PDB database, HHsearch *p*-value 1.90E−74) to the recently reported chloroplastic sulfate transporter of *Arabidopsis thaliana* (AtSULTR, PDB: 7LHV) (Wang et al., 2021). To our knowledge, no experimental prokaryotic sulfate transporter analog structure currently exists. To alleviate this situation, we obtained the high-confidence modeled structure of ACK80903 (AfSULTR) for comparative structure analysis. The AfSULTR structure exhibits a topology similar to AtSULTR: each monomer is comprised of at least 10 transmembrane (TM) helices followed by a C-terminal anti-Sigma factor antagonist (STAS) domain (Figure 3A and Supplementary Figure 3). Structure mapping shows that AfSULTR and the AtSULTR monomer have average RMSD of 3.299 angstrom (Å) (Supplementary Figure 4). Another important indication of the association between ACK80903 and membrane-anchored transporter is provided by the findings of gene co-occurrence. The analysis revealed that ACK80903 consistently co-occurred with proteins such as proteolipid membrane modulator Pmp3, Na^+^/H^+^ antiport NhaA, cation transport ATPase (P-type), AI-2E family transporter and FeoC like transcriptional regulator across the comprehensive set of *Acidithiobacillia* genomes (Supplementary Figure 4). We built the complete sulfate transporter dimer with two monomers linked by the STAS domains (anchoring on the cytosolic side of the membrane) that swap between the monomers using the dimer structure of AtSULTR (PDB 7LHV) as the dimer template (Wang et al., 2021; Figure 3B). A positively charged plane region is visible on the bottom of the TM helices, which is suggested to form electrostatic attachment to the negatively charged microbial membrane (Zhang et al., 2018b; Figure 3B). Among the TM helices of AfSULTR, the TM1–7 and TM8–14 (in reference to AtSULTR) are in a pseudo twofold symmetry, and the TM3 and TM10 arranged in a line are half helices. The crossover region between the N-termini of TM3 and TM10 leaves a crevice (the substrate-binding pocket) surrounded by residues of TM1, TM3, TM8, and TM10 at roughly the center of the TM region (Supplementary Figure 5a). These features are consistent with other secondary solute transport proteins (Lu et al., 2011; Alguel et al., 2016; Wang et al., 2019a). The STAS domain of our AfSULTR model is comprised of two α-helices and four β-strands, while the reported AtSULTR counterpart contains four α-helices and four β-strands (Wang et al., 2021). The helix dipoles of TM3 and TM10 carrying the positive electrostatic potential ends seen to orient and attract the negative electrostatic potential of oxygen anions of bound SO_4_^2–^ (Supplementary Figure 5b). Also, a conserved Arg324 (Arg393 of AtSULTR) from TM10 with positive electrostatic potential was identified to form a putative salt bridge with the bound SO_4_^2–^ (Supplementary Figure 6a). However, other surrounding residues previously shown to interact with the SO_4_^2–^ in the binding pocket in AtSULTR (e.g., Ala153, Phe391, Ser392, Tyr116, and Ser392) (Wang et al., 2021) are all missing in AfSULTR, probably due to protein family diversification. In AfSULTR, the identified surrounding residues (within 6 Å) include three leucine residues (Leu30, Leu369, and Leu379), three valine residues (Val34, Val321, and Val323), two proline residues (Pro69 and Pro71), the above-mentioned Arg324, and Thr70 (Supplementary Figure 5a). Leucine and valine contain hydrophobic side chains, which may facilitate the transport of the hydrophilic SO_4_^2–^ anion (Abdelraheem et al., 2018). Additionally, a Glu276 in AfSULTR has also been identified at approximately the same position of AtSULTR Glu347 (Supplementary Figure 6b), protonation and deprotonation of this residue is suggested to be significant for anion transport and H^+^ gradient sensing (Wang et al., 2021).

#### 2.2.2. Sulfide:quinone oxidoreductase

Sulfide:quinone oxidoreductase is a peripheral membrane protein that catalyzes the oxidation of sulfide species to elemental sulfur, belonging to the flavin disulfide reductase (DSR) superfamily (Argyrou and Blanchard, 2004). We find that the protein ortholog of *Acidithiobacillia* under accession ACK80497 putatively represents a novel unresolved SQR-like enzyme, which has a top 1 hit score to human SQR (PDB: 6OIB) (Landry et al., 2019) with 22.51% identity (86% query coverage, *E* value 1e−23). In comparison, ACK80497 shares only 47% query coverage (22.63% identity, *E* value 0.002) with the structure-available SQR homolog of *A. ferrooxidans* (PDB: 3T2Y). The prediction of ACK80497 shows the tandem Rossmann fold repeats commonly seen in the DSR superfamily topology (Argyrou and Blanchard, 2004), with RMSD of 1.992 and 2.207 Å to 6OIB and 3T2Y, respectively (Figure 3C and Supplementary Figure 7). We compared and identified the conserved triad of active residues in AfSQR-ACK80497, Cys127, Cys158, and Cys331 (equivalent to Cys128, Cys160, and Cys356 of AfSQR-3T2Y, and Cys201, Cys379 of human SQR-6OIB) (Supplementary Figure 8). Mutation of these residues was reported to lead to 70∼100% loss of activity (Griesbeck et al., 2002; Cherney et al., 2012). The active site of SQR includes a flavin adenine dinucleotide (FAD) cofactor that accepts and transfers electrons from sulfide species to ubiquinone. However, we failed to identify equivalent residues that were previously shown to bond with the cofactor FAD [e.g., Thr11, Gly12, Ser34, Ala78, Ile302, Gly322, Phe357, and Lys391 in AfSQR-3T2Y (Cherney et al., 2012)], suggesting the existence of a novel ligand-protein interaction diagram in the AfSQR-ACK80497 model. To analyze the putative ligand-protein contact, a classical MD simulation for 15 ns of AfSQR-ACK80497 was performed (Supplementary Figure 9), which showed that Met10, Ala39, His43, Gln46, Val81, Lys157, Gly298 of AfSQR-ACK80497 form strong (>50.0% occurrence) direct hydrogen bonds with atoms (e.g., O and N) of the cofactor FAD, while Val44, Ser107, Glu164 of AfSQR-ACK80497 contacts with FAD via water bridges (>50.0% occurrence) (Supplementary Figure 9). However, the actual functions of these residues still require further experimental confirmation. Another important indication of the association between ACK80497 and sulfur metabolism is provided by the findings of gene co-occurrence. The analysis revealed that ACK80497 consistently co-occurred with two specific proteins, namely the sulfur carrier protein TusA and the sulfur reduction DsrE/DsrF/DsrH family protein, across the comprehensive set of *Acidithiobacillia* genomes (Supplementary Figure 9).

#### 2.2.3. Ferrous iron transporter (FeoB)

**FIGURE 4:**
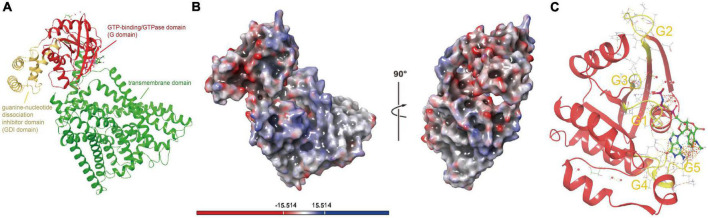
Structure visualizations of *Acidithiobacillia* ferrous iron transporter (AfFeoB), iron oxidase and ferrochelatase. **(A)** The overall structure of AfFeoB monomer that consists of G domain, GDI domain, and transmembrane domain. **(B)** Electrostatic potential surfaces of the overall AfFeoB monomer calculated with adaptive Poisson-Boltzmann solver (APBS), which is rotated 90° rightward as indicated to reveal the negatively charged enriched region of AfFeoB that putatively binds the ferric cation. **(C)** The archetypical GTPase motifs G1–G5 (shown with yellow color) in the GTPase domain of AfFeoB that flank the nucleotide-binding pocket.

Although ferrous iron is one of the primary energy substrates for *Acidithiobacillia*, we still have little knowledge about the uptake process of this substrate. The protein represented by ACK79582 (Genbank accession) encodes a membrane protein FeoB responsible for ferrous iron transport in 94.6% of tested *Acidithiobacillia* (AfFeoB) whose genomic location is highly conserved among *Acidithiobacillia* (Supplementary Figure 10). ACK79582 shows only 33% coverage and 35.61% identity (HHsearch *p*-value 3.1E−30) to the PDB hit with top 1 score (PDB 3LX5, NFeoB from *Streptococcus thermophilus*). ACK79582 was then used for structure modeling. The predicted structure of *Acidithiobacillia* (AfFeoB, represented by ACK79582) contains a N-terminal GTP-binding/GTPase domain (G domain, residues 1–169) that shows the canonical G protein fold (a six-stranded β-sheet surrounded by six α-helices), followed by the guanine-nucleotide dissociation inhibitor (GDI) domain (residues 170–257) that consists of a four-helix bundle, which links the GTPase domain and the transmembrane domain (residues 258–766) (Figure 4A). A negatively charged enriched region was identified on the surface of AfFeoB (Figure 4B), which is suggested to bind the ferric cation. We identified in the GTPase domain of AfFeoB the archetypical GTPase motifs G1–G5 that flank the nucleotide-binding pocket (Figure 4C and Supplementary Figure 11). These motifs are significant for nucleotide (GTP/GDP) recognition, orientation and reaction catalysis (Scheerer et al., 2008; Guilfoyle et al., 2009). We next compared the residues surrounding the nucleotide-binding site of AfFeoB and other reported FeoB structures (Supplementary Figure 12, key conserved residues highlighted with blue rectangles), which identified in AfFeoB the conserved residues Pro10 and Pro56 as essential for maintaining main-chain conformation and affinity for GTP and GDP (Eng et al., 2008), Asp54 and Gly57 that hydrogen-bond with the oxygen of the nucleotide γ-phosphate, Asn11 and Asn115 that contact with the GDP molecule, Asp118 associated with specificity toward the guanine base (Eng et al., 2008), as well as Ala145 and Ser150 in the G5 motif that modulate affinity and release rate of GDP (Guilfoyle et al., 2014a,b). Consistently, the structure of the transmembrane domain of AfFeoB was identified as an analog of the reported concentrative nucleoside transporter vcCNT (PDB 3TIJ) with TM-score 0.59 and RMSD 4.215 Å (Supplementary Figure 13; Johnson et al., 2012). Like vcCNT, the transmembrane domain of AfFeoB displayed an overall twofold pseudo-symmetry topology and contained two conserved nucleotide-binding residues, Glu580 and Ser682 (refer to Glu332 and Ser371 at positions similar to that of vcCNT) (Johnson et al., 2012).

#### 2.2.4. Iron oxidase

**FIGURE 5:**
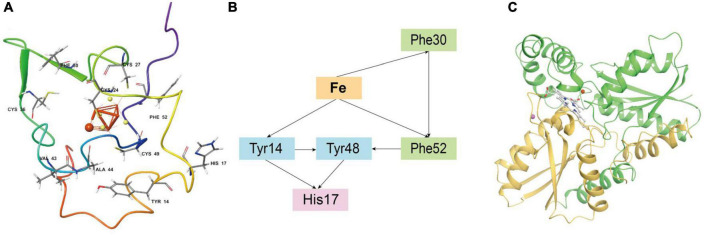
**(A)** The overall structure of *Acidithiobacillia* iron oxidase (Iro) with key conserved cysteine residues and aromatic residues that ligate/stabilize the [Fe(4)S(4)] cluster are shown in ball and stick format, and the free ferrous iron ion is represented by a red sphere. **(B)** Putative electron transfer pathway(s) in Iro identified by Emap using the structure from the last frame of MD simulation. **(C)** The overall topology of *Acidithiobacillia* ferrochelatase (ACK80603) monomer that is comprised of two similar domains (shown with yellow and green colors, respectively). The substrate protoporphyrin molecule is shown in ball and stick format. A free ferrous iron ion and a free magnesium ion are represented by red and pink spheres, respectively.

Iron oxidase (Iro, represented by Genbank ACK79288) in *Acidithiobacillia* is a key protein of the iron respiratory chain that oxidizes ferrous iron, and is closely linked with the biohydrometallurgy efficiency (Zeng et al., 2008). However, the experimental crystal structure of the Iro protein is still lacking. Residues 1–48 of ACK79288 were predicted by SignalP (Almagro Armenteros et al., 2019) to be a TAT(Tat/SPI) type signal peptide (Supplementary Figure 14) and, therefore, were removed before structure modeling. After modeling, we obtained a high-confidence overall structure of the Iro protein, a side of which was found to be mainly positively charged, forming a putative microbial membrane-bound region (Supplementary Figure 15). The [Fe(4)S(4)] cluster is in ligation with four cysteine residues (Cys24, Cys27, Cys36, and Cys49), located in the center of the protein (Figure 5A), similar to other HiPIP family proteins (Nogi et al., 2000; Ohno et al., 2017; Kawakami et al., 2021). The [Fe(4)S(4)] cluster is surrounded by the aromatic residues Tyr14, Phe30, and Phe52 (Figure 5A), which have been experimentally proven to stabilize the [Fe(4)S(4)] cluster in acid environments (Agarwal et al., 1995; Zeng et al., 2010). Tyr14 especially forms a hydrophobic barrier against solvent attack and mediates electron transfer, substitutions of which may result in protein malfunction (Iwagami et al., 1995). Classical MD simulation (15 ns) reveals that the free ferrous iron to be oxidized is captured and stabilized by Iro mainly through metal coordination effect of atoms from the [Fe(4)S(4)] cluster and ionic interactions from residues Cys24, Val43, and Ala44 (Supplementary Figure 16). We further applied Emap (Tazhigulov et al., 2019) to identify putative electron transfer pathway(s) in Iro (Figure 5B), which indicated probable electron hopping pathways from ferrous iron to phenylalanine (Phe30 and Phe52), tyrosine (Tyr14 and Tyr48) and histidine (His17) residues.

#### 2.2.5. Ferrochelatase

Ferrochelatase of *Acidithiobacillia* (presented in 98.4% of tested genomes, represented by Genbank ACK80603), involved in cofactor heme biosynthesis, is a membrane-bound protein that catalyzes the insertion of ferrous iron into protoporphyrin IX to form protoheme IX (heme). Its eukaryotic analog is encoded by the nuclear DNA and expressed in the cytoplasm, followed by translocation to the inner mitochondrial membrane, with the active site turned to the mitochondrial matrix (Sellers et al., 2001). In our study, we found that ACK80603 has top 1 hit score to human mitochondrial ferrochelatase (PDB: 2PO7, identity 27.19%, HHsearch *p*-value 1E−62). After modeling, we obtained a high-confidence structure of ACK80603 with an RMSD of 2.182 Å to human mitochondrial ferrochelatase (PDB: 2PO7), which exhibits typical ferrochelatase topology (Sellers et al., 2001), namely a monomer with two similar domains (Figure 5C, shown in green and yellow colors, respectively), each containing a Rossmann fold with a four-stranded parallel β-sheet surrounded by α-helices. The two domains are connected by a loop from residues 218–227. We predict a positively charged region in the protein surface that putatively interacts with lipid membranes (Supplementary Figure 17a), while negatively charged residues are enriched in the protoporphyrin binding pocket face (Supplementary Figure 17b). A previous study generated a reaction model for ferrochelatases based on the data of human ferrochelatase (Sellers et al., 2001), and we attempted to match the critical residues in our structure (Supplementary Figure 18):

(1) The conserved carboxylate residues Asp273, Glu276, and Glu280 corresponding to Asp340, Glu343, and Glu347 of human mitochondrial ferrochelatase (PDB 2PO7) putatively form a conduit connecting the active site pocket to the enzyme exterior and participate in proton abstraction from porphyrin (Supplementary Figure 18). Replacements of these residues are experimentally proven to hinder the proton abstraction, resulting in no product (heme) formation though the enzyme still binds with protoporphyrin (Sellers et al., 2001);
(2) Ferrous iron is transported from the exterior of the protein via residues Trp163 and Tyr129 (equivalent to Trp227 and Tyr191 of PDB 2PO7) to the site of metalation at residues Arg102 and Tyr103 (equivalent to Arg164 and Tyr165 of PDB 2PO7) centrally located in the active site pocket (Supplementary Figure 18), whose role for metalation have been confirmed by mutant tests (Sellers et al., 2001);
(3) The central catalysis residue His195 (refers to His263 of PDB 2PO7) on the opposite side of Arg102 and Tyr103 acts as the proton-acceptor of porphyrin that initializes metalation in conjunction with proton abstraction. All mutants at His263 (PDB 2PO7) have no measurable enzyme activity (Sellers et al., 2001). Additionally, Trp243 (Trp310 in PDB 2PO7) is involved in saddling of the porphyrin during catalysis (Shi et al., 2006).

Regarding comparisons with microbial ferrochelatase, we also found equivalent residues to Arg115, Tyr123, and Ser130 of *Saccharomyces cerevisiae* ferrochelatase (PDB 2HRE), namely Arg53, Tyr61, and Ser70 in our structure, which putatively form an interaction network with the protoporphyrin substrate as observed previously (Stroupe et al., 2003). Finally, His195 and Glu276 in our structure are located at a position similar to the highly conserved residues His183 and Glu264 in *Bacillus subtilis* ferrochelatase (PDB 2Q2N) that putatively facilitate the insertion reaction of the metal ion into protoporphyrin IX (Hansson et al., 2007). While the conserved active site residues Pro268 and Trp243 (refer to Pro255 and Trp230 of PDB 2Q2N), located in a loop, putatively modulate the regio-specificity of porphyrin binding (Karlberg et al., 2008). MD-based protein-ligand interaction analysis further illustrates that hydrogen-bonding from Arg53, Ser57, Tyr61, Trp65, Ser70, Val275, Glu276, and hydrophobic contact/Pi-Pi stacking from Phe27 take part in stabilization of porphyrin molecule (Supplementary Figure 19). Besides, metadynamics analysis shows that ferrous iron stays at the protein conduit without leaving the protein throughout the simulation (20 ns, with ferrous iron firstly placed at the protein entrance position). Free-energy profile of the two collective variables (CVs) depicted in Supplementary Figure 20 that measure the distances of ferrous iron to metalation residue Tyr103 (C1) and protoporphyrin (N1) throughout simulation (20 ns) displays a wide and deep basin, which indicates that the ferrous iron can be stably captured by the protein (Supplementary Figure 21). However, the actual roles of the above-mentioned residues require confirmation by further experimental studies.

#### 2.2.6. Functional predictions of unannotated proteins

A full 35.8% of proteins in the proteome of *Acidithiobacillia* are still labeled as “hypothetical protein” or “domain of unknown function.” This microbial “dark matter” awaits exploration, and may also be of significant relevance, especially to the biohydrometallurgy capability of *Acidithiobacillia*. Yet, it is still difficult to crack the mysteries of their functional identity by traditional methods (e.g., genetic manipulation) due to the slow growth rate of this autotrophic organism (Marchand and Silverstein, 2002). Considering that the function of a protein is ultimately defined by its structure, fortunately, many available state-of-the-art deep-learning algorithms can be utilized, such as DeepFRI, who’s ability for reliable structure-based function classification and prediction of unknown proteins have been validated (Gligorijević et al., 2021). Thus, we first used the predicted 3D structures of unannotated proteins in the proteome of *Acidithiobacillia* as inputs for DeepFRI (Gligorijević et al., 2021) to perform function prediction. Results show that 93.6% (1,055/1,127) of the unannotated proteins in *Acidithiobacillia* could be assigned structure-based gene ontology (GO) term predictions of cellular components (Supplementary Table 2), in which the GO terms cytoplasm (GO:0005737, 29.0%, 306/1,055) and membrane (GO:0016020, 25.8%, 272/1,055) accounted for the largest proportions. A total of 91.3% (1,029/1,127) of unannotated proteins in *Acidithiobacillia* could be assigned structure-based GO term predictions of molecular function (Supplementary Table 2), in which the GO terms cellular metabolic process (GO:0044237, 20.0%, 206/1,029) and heterocyclic compound binding (GO:1901363, 10.3%, 106/1,029) accounted for the largest proportions. About 91.8% of these hypothetical proteins have confident scores above the DeepFRI significance cut-off score of 0.5 (Supplementary Table 2; Gligorijević et al., 2021), indicating that the predictions are reliable.

Obtaining the general GO-term prediction is only the initial step toward the final characterization of targeted unknown proteins. Other recently published advanced algorithms, such as CHARMM-GUI LBS Finder and Refiner (Guterres et al., 2021) that performs local structure alignment and virtual screening, provide additional tools to identify the putative substrate(s) for an unknown protein using its structural information. For instance, ACK77828 (conserved hypothetical protein) was predicted to be involved in cellular nitrogen compound metabolic process (GO:0034641, score 0.99) by DeepFRI (Gligorijević et al., 2021; Supplementary Table 2). Consistent with this, LBS Finder and Refiner (Guterres et al., 2021) predicted the most probable substrate of ACK77828 is a nitrogen-containing compound, namely (2R)-2-amino-3-hydroxysulfanyl-propanoic acid (C_3_H_7_NO_3_S, CSO), and CSO was predicted by LBS Finder and Refiner (Guterres et al., 2021) to be bound by ACK77828 in a similar pattern with the transcriptional regulator SarZ (PDB: 3HRM) (Supplementary Figure 22).

**FIGURE 6:**
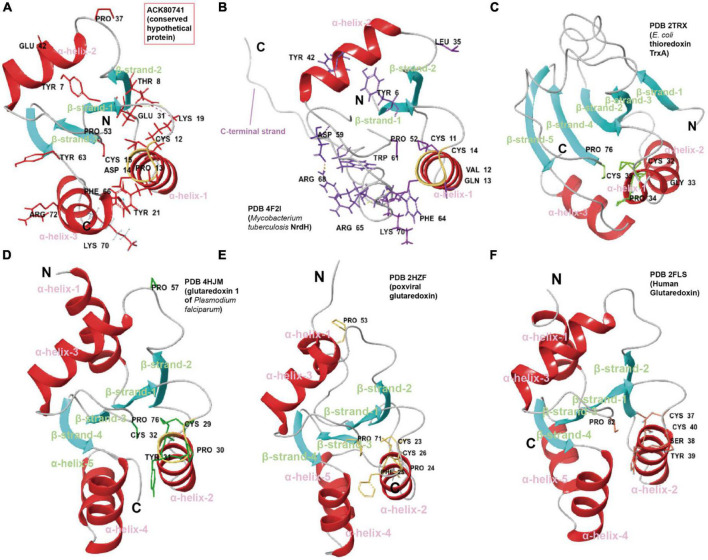
Cartoon representations and suggested key residues of **(A)** ACK80741, **(B)** *Mycobacterium tuberculosis* NrdH (PDB 4F2I), **(C)** thioredoxin from *Escherichia coli* TrxA (PDB 2TRX), **(D)** glutaredoxin 1 of *Plasmodium falciparum* (PDB 4HJM), **(E)** poxviral glutaredoxin (PDB 2HZF), and **(F)** human glutaredoxin (PDB 2FLS). The coloring is based on secondary structure (helix, red; sheet, blue; and loop, gray).

Robust structure comparison, fold recognition, catalytic site configuration and evolutionary analysis of residues can also be useful during such functional inference. In another case, ACK80741 (conserved hypothetical protein) was given the GO function prediction, disulfide oxidoreductase activity (GO:0015036, score 0.70) by DeepFRI (Gligorijević et al., 2021). Consistent with this, structure comparisons of ACK80741 model (confidence 0.92) with crystalized disulfide oxidoreductase (DSR) family proteins including glutaredoxin (Grx), thioredoxin (Trx), and NrdH show that ACK80741 possesses the combined features of reported DSR proteins (Figure 6). ACK80741 exhibits the typical Grx/Trx fold, consisting of a core of four (anti)parallel β-strands flanked by α-helices. In ACK80741, we observed that Lys19 (α-helix-1) forms a hydrogen bond and salt bridge with Thr8 (β-strand-1) and Glu31 (β-strand-2), respectively, and Tyr21 (α-helix-1) and Phe66 (α-helix-3) are involved in an aromatic-aromatic interaction. These cross-helix/strand interactions may be significant for overall structure stability (Lanzarotti et al., 2011) and some of them seem to be unique to ACK80741. Another important indication of the association between ACK80741 and redox hemostasis is provided by the findings of gene co-occurrence. The analysis revealed that ACK80741 consistently co-occurred with respiratory proteins (i.e., respiratory chain assembly protein Aim24 and cytochrome B561), sulfur oxidation *sox* operon protein DUF302 and the stress response protein, Copper binding periplasmic protein CusF, across the comprehensive set of *Acidithiobacillia* genomes (Supplementary Figure 23). ACK80741 seems to be closer to the NrdH clade in the phylogenetic tree (Supplementary Figure 23). Like NrdH, ACK80741 does not possess the additional N-terminal β-strand/α-helix present in Grx and Trx structures, while ACK80741 also lack the long C-terminal strand present in NrdH, and the loop regions of ACK80741 are generally shorter than those of Trx. Furthermore, the short α-helices 4 and 5 in Grx structures seem to have merged into a relatively long α-helix in ACK80741 (α-helix-3) (Figure 6). We recognize the active site cysteine pair motif (C12-P-D-C15) located in the loop connecting the first β-strand to the second α-helix in ACK80741, which is different from NrdH (CVQC), TrxA (CGPC), and Grx (CPY(F)C) in the residues between the two cysteines (Supplementary Figure 23). These residues may affect the redox potential and pKa value of protein (Chivers et al., 1997). In addition to the common turn-inducing Pro53 at the start of β-strand 3, ACK80741 possesses another cis-proline, Pro37 (corresponding to Pro53 of poxviral glutaredoxin, PDB 2HZF) at the start of the third α-helix, which has been shown to be uniquely conserved in orthopoxvirus Grx orthologs (Bacik and Hazes, 2007). ACK80741 also possesses conserved Arg72 and Tyr63, counterparts of MtNrdH Arg68, Trp61 (PDB 4F2I), which are suggested to form a cation-Pi interaction. Lys70, referring to the kink-causing Lys70 of MtNrdH, and Tyr7 that corresponds to Tyr6 of MtNrdH (Figure 6; Phulera and Mande, 2013). Further, classical MD simulation (15 ns) reveals that Arg50, Ala52, Thr65, Asp14, Ser9, and Glu68 are important residues involved in substrate (glutathione, GSH) binding and interaction (Supplementary Figure 24). Still, the actual functions of the proteins mentioned above require experimental confirmation.

## 3. Discussion

In this study, we conducted protein structure predictions using AI for *Acidithiobacillia* bacteria, which is commonly employed in biohydrometallurgy. The predictions covered the entire pan-proteome of *Acidithiobacillia*. These structural insights are highly valuable for future research focused on understanding mechanisms and designing proteins. This study builds upon our laboratory’s prior investigations into the unique characteristics and applications of *Acidithiobacillia* bacteria (Li et al., 2019; Yang et al., 2020; Tao et al., 2021).

Although continuous efforts have been made to upgrade experimental approaches for protein structure determination, the speed of discovering known structures still lags behind that of sequencing data (Goodsell et al., 2020). Structures of membrane proteins (accounting for only ∼1% PDB entries) are particularly difficult to resolve since they tend to denature and aggregate during purification once removed from their native membrane environment (Bill et al., 2011). In our study, we found that 23.9% of hypothetical proteins in the proteome of *Acidithiobacillia*, as predicted by DeepFRI (Gligorijević et al., 2021), were to be located in the cellular membrane component (highlighted with blue color in Supplementary Table 2). The membrane-related proteins play a significant role in various transportation processes and the electron transfer chain, closely associated with iron/sulfur utilization (Yarzábal et al., 2002; Castelle et al., 2008). The elucidation of their structures may shed light on the molecular mechanisms underlying biohydrometallurgy processes in the extreme acidophile *Acidithiobacillia*. Our results demonstrate that the overall prediction reached an average confidence of 0.76 regarding structure prediction of the membrane proteins from *Acidithiobacillia*. This indicates that even for protein classes with limited examples for training datasets, we are able to confidently predict their structures. Additionally, we have highlighted the molecular details of certain membrane proteins (e.g., sulfate transporter) in our case studies. These achievements contribute to why AI-driven accurate protein prediction was selected by the journal *Science* as the top breakthrough of the year 2021 (Thorp, 2021). In our study, more than half of these predictions were of high quality, which is a significant improvement compared to previous studies with less than 40% accuracy (Zhang and Skolnick, 2004).

Additionally, we found that the prediction confidence was independent of the protein sequence length (data not shown), suggesting the capacity of AlphaFold2 (Senior et al., 2020) and RoseTTAFold (Baek et al., 2021) to maintain prediction accuracy, even during the structural prediction of large proteins. This was likely due to the innovation in combining predictions from multiple discontinuous regions to produce an overall structure (Baek et al., 2021), which has outperformed many other modeling programs like Swiss-Model (Waterhouse et al., 2018). These predicted protein structures, when combined with other structure-based analyses, can provide valuable insights into the molecular mechanisms of target proteins and generate scientific hypotheses, including the identification of uncharacterized reaction sites or novel substrate interaction diagrams, as demonstrated in previous studies (Baek et al., 2021; Humphreys et al., 2021; Tunyasuvunakool et al., 2021) and this study (see section “2.2. Highlight of predicted structures”).

Although acquiring protein sequence and structural data has become relatively easy, accurately predicting the function of unannotated proteins remains a challenge. In fact, less than 0.8% of the sequences in the UniProt Consortium (2015) have been experimentally characterized and manually annotated in SwissProt (Boutet et al., 2007). Additionally, about 80% of poorly annotated sequences in current databases do not have analogs with similar functions, and 25% of them have no identifiable analogs with a query identity greater than 30% (Zhang et al., 2017). This makes it difficult to perform annotations using traditional homologous transfer approaches. In our study, 35.8% of the proteome from *Acidithiobacillia* consisted of proteins with unknown functions. We propose that the 3D structure of proteins may offer a possible solution to this problem, as the majority of protein domains tend to adopt unique, ordered, and recognizable 3D fold conformations (Das et al., 2015). We were pleased to find that several advanced structure-based high-throughput annotation algorithms are emerging to tackle this challenge, including DeepFRI (Gligorijević et al., 2021), LBS Finder and Refiner (Guterres et al., 2021), CATH (Das et al., 2015), and COFACTOR (Zhang et al., 2017). These algorithms provide valuable information for detailed structure comparison, fold recognition, catalytic site identification, *in silico* reaction simulation, and experimental verification. Some of these algorithms were highlighted in our results (see section “2.2.6. Functional predictions of unannotated proteins”). They leverage structural information, such as the spatial position of amino acids, dihedral angles, the contact matrix representing spatial distances between amino acids, and sub-structure frequency, which have been shown to outperform previous sequence-based prediction methods (Gligorijević et al., 2021). Additionally, gene correlation networks can predict the functions of previously unknown genes based on the functions of adjacent genes (Ma et al., 2018). Other structure-based strategies for enzyme functional characterization have also been demonstrated in previous studies. For example, Zhao et al. (2013) applied large-scale metabolite docking of available 3D protein structures against the KEGG metabolite library and successfully characterized a series of enzymes of unknown functions. Likewise, Hitchcock et al. (2013) proposed the substrate profiles of uncharacterized enzymes by docking metabolites to modeled structures. Finally, Mokrushina et al. (2020) combined structural information with the quantum mechanics/molecular mechanics (QM/MM) method to uncover the catalytic mechanism of an immunoglobulin with novel functionality, which can guide the artificial evolution of valuable enzymes. These strategies significantly expand the possibilities for characterizing and applying unknown proteins.

## 4. Materials and methods

**FIGURE 7:**
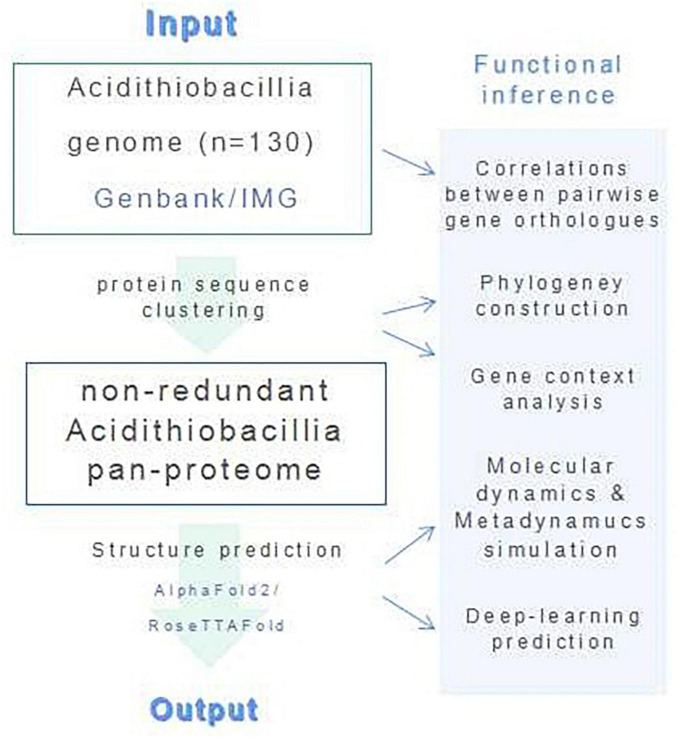
A graphical workflow of the computational processes.

We have combined protein structural and phylogenetic analyses in this study (Figure 7).

### 4.1. Obtaining sequence of *Acidithiobacillia*

Protein sequences and protein-encoding gene sequences for the proteome of all available *Acidithiobacillia* isolates (*n* = 129) were downloaded from Genbank (Benson et al., 2018). All protein sequences within the proteome were first manually checked for the presence of ambiguous residue codes (B, J, O, U, Z, or X). Sequence(s) containing these ambiguous residue codes was discarded. Orthofinder v.1.0 with default parameters was used for protein sequence clustering (Emms and Kelly, 2019). The protein sequences were reannotated with eggnog mapper v.2.0 (Cantalapiedra et al., 2021). The size of the *Acidithiobacillia* pangenome was extrapolated by implementing an power law regression function, *Ps* = κnγ, using a built-in program of the BPGA v.1.0 (Chaudhari et al., 2016),^1^ in which *Ps* represents the total number of non-orthologous gene families within its pangenome, *n* represents the number of tested strains, and both κ and γ are free parameters. An exponent γ of <0 suggests the pangenome is “closed,” where the size of the pangenome reaches a constant value as extra genomes are added. Conversely, the species is predicted to harbor an “open” pangenome for γ values between 0 and 1. In addition, the size of the core genome was extrapolated by fitting into an exponential decay function, *Fc* = κcexp(−n / τc), with a built-in program of the BPGA pipeline (Chaudhari et al., 2016), where *Fc* is the number of core gene families, and κc, τc are free parameters. To construct the gene ortholog association network, correlations between pairwise gene orthologs that were present in more than half of the genome were calculated using the CoNet methods in Cytoscape v.3.9.1.^2^ Only edges with a significant correlation higher than 0.7 (*p* < 0.05) were retained for network construction. The COG functional categories were assigned by eggNOG-mapper v2 (–evalue 0.001 –score 60 –pident 40 –query_cover 20) after annotation of the query sequences against the COG database (Galperin et al., 2021). We applied Clustal Omega (Sievers and Higgins, 2018) for multiple sequence alignments (MSAs). Enzyme Function Initiative-Genome Neighborhood Tool (EFI-GNT) (Gerlt, 2017) was used to analyze the gene context in genomes. We used SignalP v.5.0 (Almagro Armenteros et al., 2019)^3^ for signal peptide prediction and SOSUI (Hirokawa et al., 1998)^4^ for transmembrane region predictions.

### 4.2. Proteome-scale structure prediction and analysis

We configured the local version of AlphaFold2 (Senior et al., 2020) and RoseTTAFold (Baek et al., 2021) on our laboratory’s computation resource, a Dell PowerEdge R940xa server with four Intel Xeon Platinum 8260 processors (total of 148 cores), 1 TB of RAM, installed with Ubuntu 18.04.6 distribution, python 3. Prediction of protein 3D structure was conducted for all checked sequences within the pan-proteome of *Acidithiobacillia* through the local installation. The modeling analysis is generally comprised of six steps: (1) Generate MSAs. (2) Predict secondary structure for HHsearch run. (3) Search for templates. (4) Predict distances and orientations. (5) Perform modeling. (6) Pick final models. The prediction confidences were estimated by multiplying residue-wise accuracy using DeepAccNet (Hiranuma et al., 2021). We applied Visual Molecular Dynamics (VMD) software v.1.9.4 (Humphrey et al., 1996) for structural analysis, visualization, and graphics production. Electrostatic potential was calculated with an adaptive Poisson-Boltzmann solver (APBS) (VMD APBS Plugin, version 1.3.1). TM-align program (Zhang and Skolnick, 2005) was used for structure comparisons. For substrate catalyzing proteins, we applied AutoDock Vina v1.2.1 (Trott and Olson, 2010) to dock the ligand into the predicted structure in reference to its PDB template. All structural predictions generated in this study are available to the community via https://doi.org/10.6084/m9.figshare.19093109.v3.

### 4.3. Molecular dynamics and metadynamics simulations

Molecular dynamics simulations for the protein-ligand complex were performed using the Desmond Molecular Dynamics System, version 3.6 (D. E. Shaw Research, New York, NY, 2008), with OPLS_2005 force field. We built the simulation system with periodic boundary conditions (PBC), which placed all molecules of the protein-ligand complex in an orthorhombic periodic boundary box with water solvent molecules, together with sodium or chloride ions to balance the systems. Before the production phase, we performed equilibration and energy minimization with the default workflow of Desmond. We conducted MD simulations in an NPT ensemble at a temperature of 300 K and an atmospheric pressure of 1.01325 bar. We integrated the equations of motion with the RESPA integrator, which applied an inner time step of 2.0 fs for bonded and non-bonded interactions within the short-range cut-off and an outer time step of 6.0 fs was used for non-bonded interactions beyond the cut-off. We calculated long-range electrostatic interactions with the Particle-mesh Ewald (PME) method applying a grid spacing of 0.8 A. Additionally, bonds to hydrogen atoms were constrained with the M-SHAKE method. After energy minimization, all molecules were subjected to the final production run for 15–20 ns. The last frame of MD simulation was used as input model for the following metadynamics simulation process using the Desmond Molecular Dynamics System, version 3.6 (D. E. Shaw Research, New York, NY, 2008), for a total of 20 ns. For the metadynamics distance CVs, the Gaussian width was set to 0.05 Å. The starting height of the Gaussian potential was set to 0.03 kcal/mol, and the Gaussians were deposited every 0.09 ps. The simulation was conducted at 300 K and 1.01325 bar pressure. RESPA integrator was applied with a time step of 2.0 fs, and short-range cut-off radius was defined at 9 Å. Electron transfer across the targeted protein was calculated using the structure from the last frame of MD simulation with eMap (Tazhigulov et al., 2019) (default parameters), which applied the graph theory to predict electron tunneling through electron transfer active moieties.

### 4.4. Phylogenetic tree construction

Phylogenetic tree based on protein sequences was built using PhyML (Guindon et al., 2010) with the Maximum Likelihood (ML) method and 1,000 bootstrap replicates, followed by visualization with iTOL (Letunic and Bork, 2021). Sequences were aligned with MUSCLE (Edgar, 2004) and trimmed with Gblocks (Talavera and Castresana, 2007) prior to tree construction.

## 5. Conclusion

In this study, by utilizing the advanced AI-driven method, we generated for the first time reliable full-chain structure predictions for the pan-proteome of *Acidithiobacillia*, the model strain for biohydrometallurgy. The median of model confidences was 0.80, and proteins assigned to COG F (nucleotide transport and metabolism), COG H (coenzyme transport and metabolism), and COG J (translation, ribosomal structure and biogenesis) had the highest average confidences. For the convenience of further analyses, the predictions are freely available to the community. In addition, several case studies on structures of conserved sulfur and iron utilization proteins (e.g., sulfate transporter and iron oxidase) that illustrate the effect of high-accuracy predictions are also supplemented. Finally, for the 35.8% unannotated proteins in the proteome, we resorted to the deep-learning algorithm DeepFRI for structure-based functional predictions and successfully obtain GO terms for 93.6% of these unknown proteins. These results pave the way for a better understanding of the biological role of *Acidithiobacillia* in biohydrometallurgy applications.

## Data availability statement

The original contributions presented in this study are included in the article/Supplementary material, further inquiries can be directed to the corresponding authors.

## Author contributions

LL and LZ conceived and designed the research. CJ, ZL, DM, FL, QH, and HY analyzed the data. LL wrote the manuscript. All authors contributed to the article and approved the submitted version.

## Footnotes

1
https://sourceforge.net/projects/bpgatool/

2
https://cytoscape.org

3
http://www.cbs.dtu.dk/services/SignalP/

4
http://harrier.nagahama-i-bio.ac.jp/sosui/
