# Supplementary material for: AI-driven pan-proteome analyses reveal insights into the biohydrometallurgical properties of Acidithiobacillia
Source: Front Microbiol. 2023 Sep 7;14:1243987. doi: 10.3389/fmicb.2023.1243987 (PMC10512742; doi:10.3389/fmicb.2023.1243987)
Supplement: Supplementary file 2 [file Data_Sheet_1.PDF]

Supplementary Material

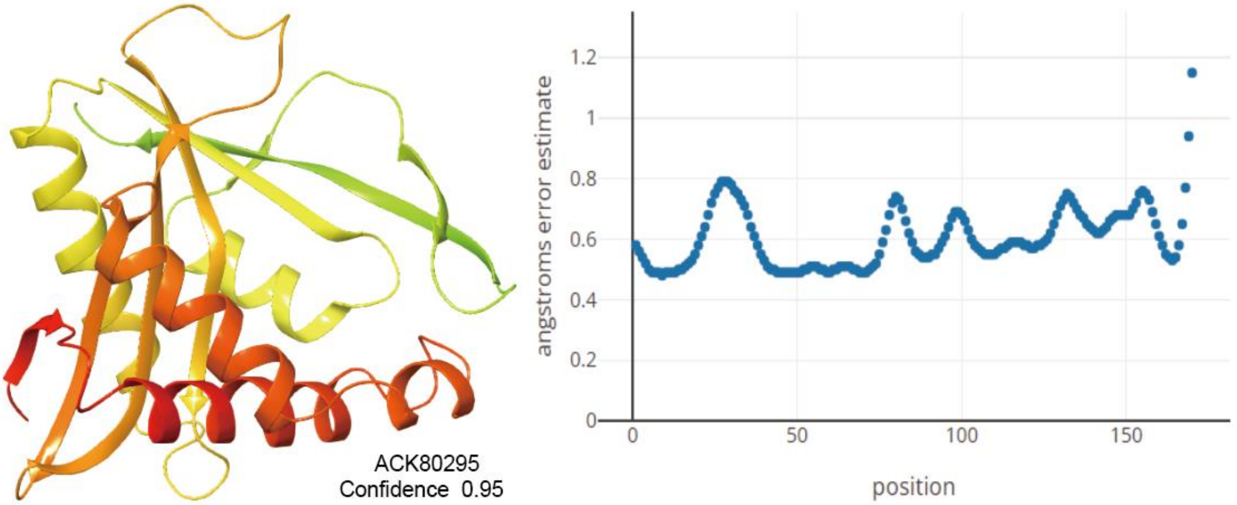

Fig. S1: The predicted model of ACK80295 (GNAT family acetyltransferase) in *Acidithiobacillia* has the highest confidence 0.95 in this study. The overall predicted structure of ACK80295 (left) and the dot plot showing angstroms error estimate per residue (right).

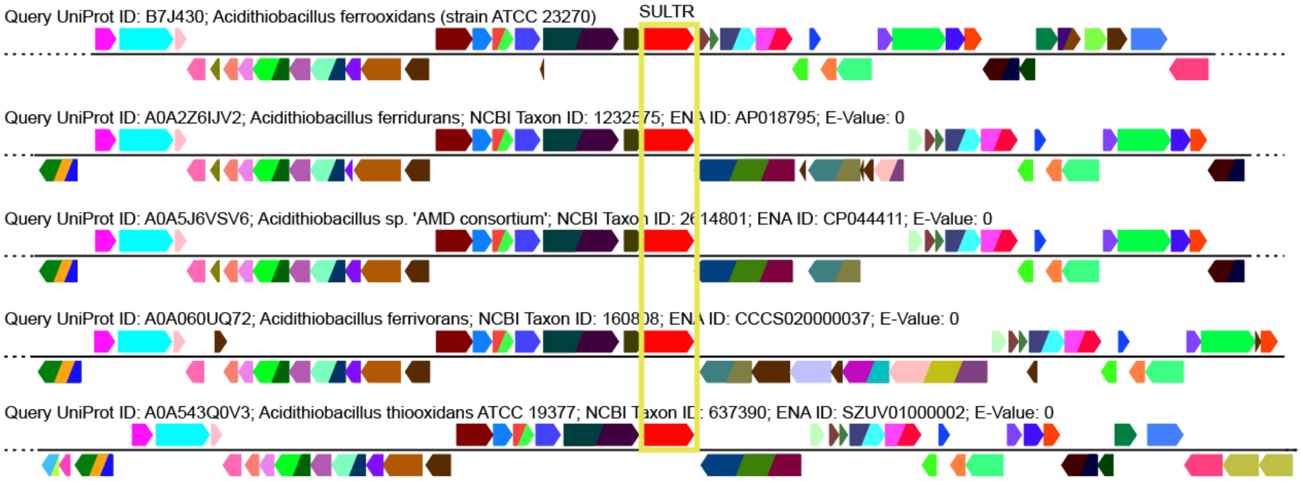

Fig. S2: Genome context comparisons of ACK80903 sulfate transporter (AfSULTR) in *Acidithiobacillia*, which suggest a conserved evolutionary relation.

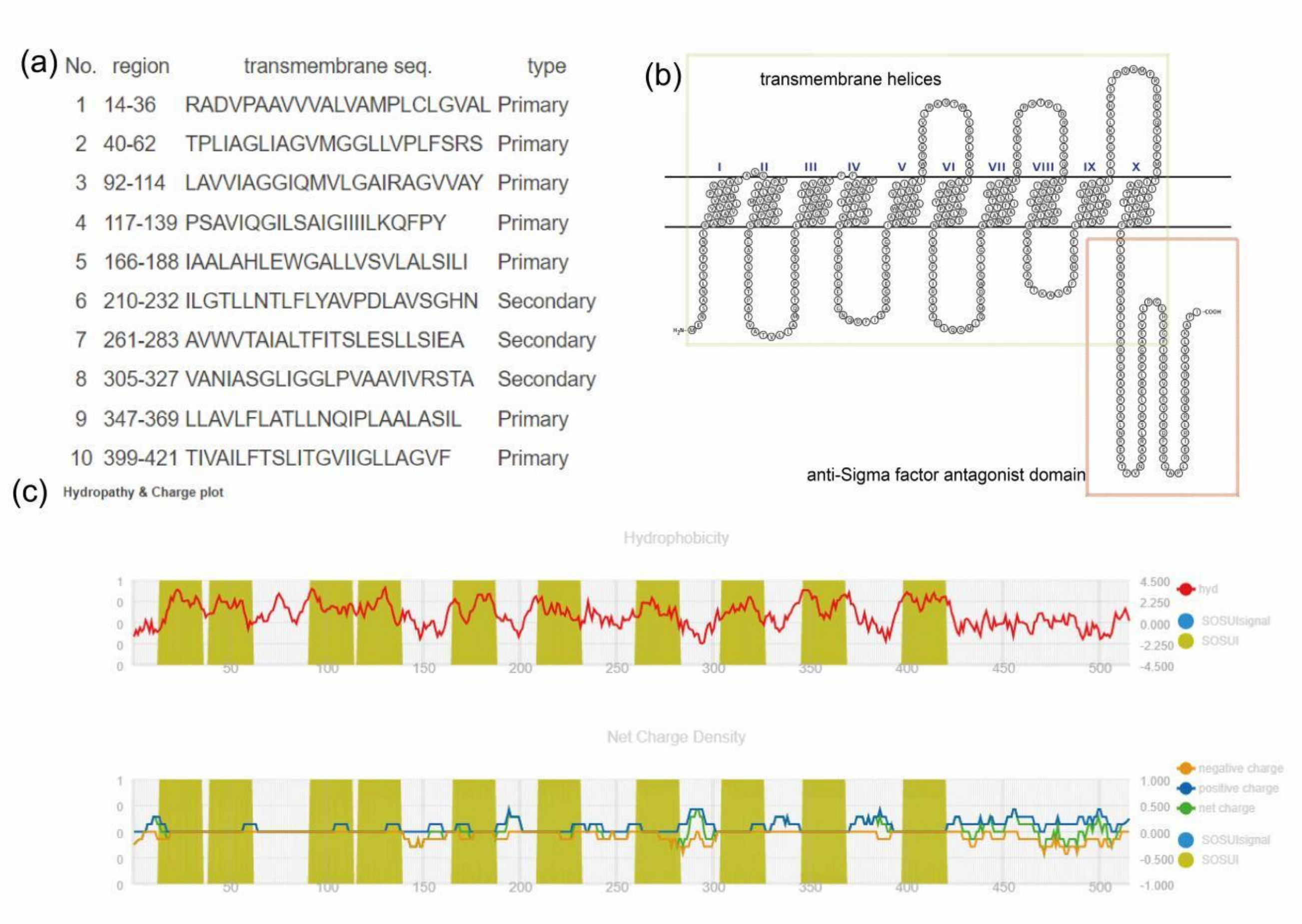

Fig. S3: Transmembrane regions of ACK80903 sulfate transporter (AfSULTR) in *Acidithiobacillia* by SOSUI. (a) Sequences segments of the prediction for transmembrane helix regions; (b) Snake like plot showing overall transmembrane regions in ACK80903; (c) Hydropathy & Charge plot showing description of hydrophobicity and charge properties of predicted transmembrane helical regions.

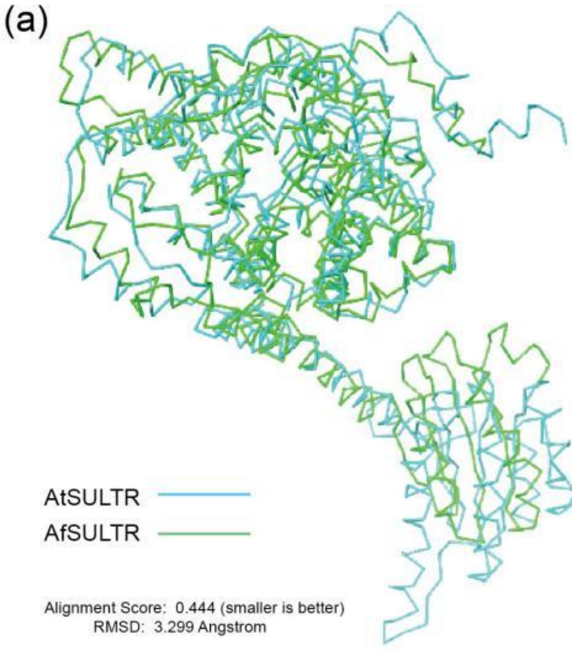

(b)

```
          ..........+.........+.........+.........
7LHV      SSA 21   70 CCHHHHHHHHCHHHHHHHHCCHHHHHHHHHHHHHHHHHHH
ACK80903  SSA 22    1 -----C-H---HHHHHC--C-C-CCHHHHHHHHHHHHHHH
7LHV          21   70 MRLVDWIDTLFPCFRWIRTYRWSEYFKLDLMAGITVGIML
ACK80903      22    1 -----M-E---NSANLS--P-F-KNLRADVPAAVVVALVA

          ..........+.........+.........+.........
7LHV      SSA 21  110 HHHHHHHHHHHCCCHHHHHHHHHHHHHHHHHHHCCCCCCCC
ACK80903  SSA 22   28 HHHHHHHHHHHCCCHHHHHHHHHHHHHHHHHHHHCCCCCCC
7LHV          21  110 VPQAMSYAKLAGLPPIYGLYSSFVPVFVYAIFGSSRQLAI
ACK80903      22   28 MPLCLGVALASGTPLIAGLIAGVMGGLLVPLFSRSQLAVS

          ..........+.........+.........+.........
7LHV      SSA 21  150 CCCHHHHHHHHHHHHHHHHHCCCCHHHHHHHHHHHHHHHHH
ACK80903  SSA 22   68 EEEHHHHHHHHHHHHH-HCC----CHHHH-HHHHHHHHHHH
7LHV          21  150 GPVALVSLLVSNALGGIADTNEELHIELAILLALLVGILE
ACK80903      22   68 GPTPAIVATVLLAMQ-TLP----SFSAF-LLAVVIAGGIQ

          ..........+.........+.........+.........
7LHV      SSA 21  190 HHHHHHCCHHHHHCCCHHHHHHHHHHHHHHHHHHHHHHHH-H
ACK80903  SSA 22  102 HHHHHHCCHHHHHHHHCHHHHHHHHHHHHHHHHHHHHHHHH
7LHV          21  190 CIMGLLRLGWLIRFISHSVISGFTSASAIVIGLSQIKY-F
ACK80903      22  102 MVLGAIRAGVVAYFFPSAVIQGILSAIGIIIILKQFPYAI

          ..........+.........+.........+.........
7LHV      SSA 21  229 HCCCCCC--CCCHH-----HHHHHHHHHHCCCCHHHHHHH
ACK80903  SSA 22  142 -CCCCCCCCCCCCCCCCCCCHHHHHHHHHHHCCHHHHHHH
7LHV          21  229 LGYSIAR--SSKIV-----PIVESIIAGADKFQWPPFVMG
ACK80903      22  142 -GFDLGEFGNQDFISAHGENTFTGVIAALAHLEWGALLVS

          ..........+.........+.........+.........
7LHV      SSA 21  262 HHHHHHHHHHHHHHHHHH-CCCCHHHHHHHHHHHHHHHHHH
ACK80903  SSA 22  181 HHHHHHHHHHHHH--CHHHHHCCC-C-HHHHHHHHHHHH
7LHV          21  262 SLILVILQVMKHVGKAK-KELQFLRAAAPLTGIVLGTTIA
ACK80903      22  181 VLALSILITWDKV--AVLRKQTWL-S-GPLMAVILGTLLN

          ..........+.........+.........+.........
7LHV      SSA 21  301 HCCC--C-CC-CC---CCCCCCC--CC--CC--CCCCC--
ACK80903  SSA 22  217 HHHHHHCCCCCCCCCCCCCC-CCCCCCHHHHHHHHCCCCCC
7LHV          21  301 KVFH--P-PS-IS---LVGEIPQ--GL--PT--FSFPR--
ACK80903      22  217 TLFLYAVPDLAVSGHNLVN-LPIIRSVADLQGMLQMPDWG

          ..........+.........+.........+.........
7LHV      SSA 21  326 CC-C-CHHHHHHHH-HHHHHHHHHHHHHHHHHHHHHHCCC
ACK80903  SSA 22  256 CCCCHHHHHHHHHHHHHHHHHHHH-HHHHHHHHHH-CCCCC
7LHV          21  326 SF-D-HAKTLLPTS-ALITGVAILESVGIAKALAAKNRYE
ACK80903      22  256 LIGSKAVWVTAIALTFITSLESL-LSIEAADKLD-VFKRR

          ..........+.........+.........+.........
7LHV      SSA 21  363 CCHHHHHHHHHHHHHHHHHHHCCCCCCCCHHHHHHHHHHCC
ACK80903  SSA 22  294 CCHHHHHHHHHHHHHHHHHHHCCCEEECCHHHHHHHHHHCC
7LHV          21  363 LDSNSELFGLGVANILGSLFSAYPATGSFSRSAVNNESEA
ACK80903      22  294 TPLDRELLGQGVANIASGLIGGLPVAAVIVRSTANVAAGA

          ..........+.........+.........+.........
7LHV      SSA 21  403 CCHHHHHHHHHHHHHHHHHCHHHHHHCCCHHHHHHHHHHHH
ACK80903  SSA 22  334 CCHHHHHHHHHHHHHHHHHCHHHHHHHHHCHHHHHHHHHHH
7LHV          21  403 KTGLSGLITGIIIGCSLLFLTPMFKYIPQCALAAIVISAV
ACK80903      22  334 RTKASAFLHGLFLLLAVLFLATLLNQIPLAALASILIFVG

          ..........+.........+.........+.........
7LHV      SSA 21  443 HHHHCHHHHHHHHHHHC-HHHHHHHHHHHHHHHHHHC-HHHH
ACK80903  SSA 22  374 HHHCCHHHHHHHHHH-CCHHHHHHHHHHHHHHHH-CCCHHHH
7LHV          21  443 SGLVDYDEAIFLWRVD-KRDFSLWTIITSTITLFFG-IEIG
ACK80903      22  374 FKLAHPSIFQRMFR-LDKSQYLPFMITIVAIL-FTSLITG

          ..........+.........+.........+.........
7LHV      SSA 21  481 HHHHHHHHHHHHHHHHHHC--CEEEEECCCCCCCCCCCCC
ACK80903  SSA 22  412 HHHHHHHHHHHHHHHHHHHHHHEEEEEEE-------------
7LHV          21  481 VLVGVGFSLAFVIHESAN--PHIAVLGRLPGTTVYRNIKQ
ACK80903      22  412 VIIGLLAGVFFVLKANYHSAIEIGRE--------------

          ..........+.........+.........+.........
7LHV      SSA 21  519 CCCCCCCCEEEEEEEE--CCECCCCHHHHHHHHHHHHHHH
ACK80903  SSA 22  438 --------CE-EEEEECCCCECCCCHHHHHHHHHHH----
7LHV          21  519 YPEAYTYNGIVIVRID--SPIYFANISYIKDRLREYEVAV
ACK80903      22  438 --------GA-AYKIALNREVIFVNKARLSHILERL----

          ..........+.........+.........+.........
7LHV      SSA 21  557 HHHHHHCCCCCCCE-EEEEECC--C---CCCCC--HHHHH
ACK80903  SSA 22  465 -------------HCCEEEEEECCCCCCCCH-HHHHHHHH
7LHV          21  557 DKYTNRGLEVDRIN-FVILEMS--P---VTHID--SSAVE
ACK80903      22  465 -------------PKGAEVILDGEHVGFIDH-DVLEVIRD

          ..........+.........+.........+.........
7LHV      SSA 21  589 HH-HHH-HHHHHHHCCEEEEE-E-C---C-HHHHHHHHHH
ACK80903  SSA 22  491 HHHH--HHC------CCCEEEEECCCCCCCCC-C-C----
7LHV          21  589 AL-KEL-YQEYKTRDIQLAIS-N-P---N-KDVHLTIARS
ACK80903      22  491 FERS--APL------REIRLREQGFDAPVLKA-P-I----

          ..........+.........+.........+.........
7LHV      SSA 21  621 CHHHHHCCCCCCCCHHHHHHHHHH
ACK80903  SSA 22      ------------------------
7LHV          21  621 GMVELVGKEWFFVRVHDAVQVCLQ
ACK80903      22      ------------------------
```

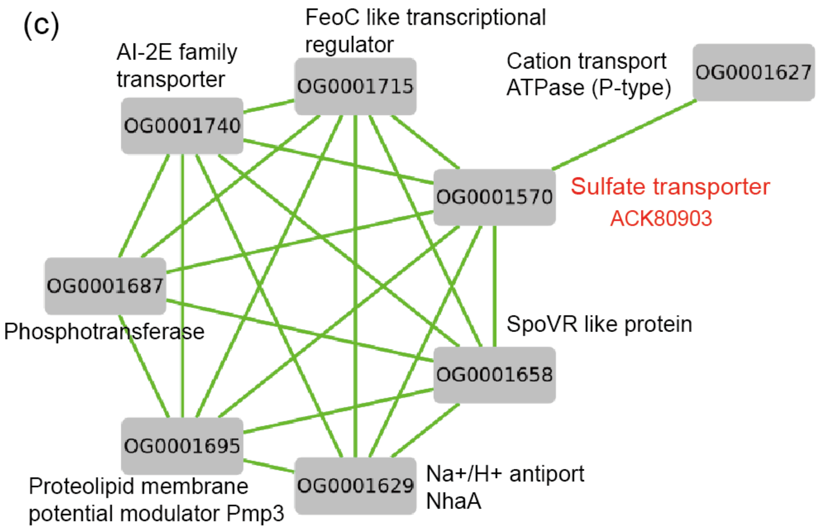

Fig. S4: (a) Structure alignment and (b) sequence alignment of ACK80903 sulfate transporter (AfSULTR) of *Acidithiobacillia* monomer and the chloroplastic sulfate transporter of *Arabidopsis thaliana* (AtSULTR, PDB: 7LHV) monomer. Secondary structure abbreviation: helix, sheet, and coil are abbreviated as H, E, and C. (c) The gene co-occurrence sub-network showing the neighbors of protein ACK80903 (spearman *cor* >0.7, *p*<0.05).

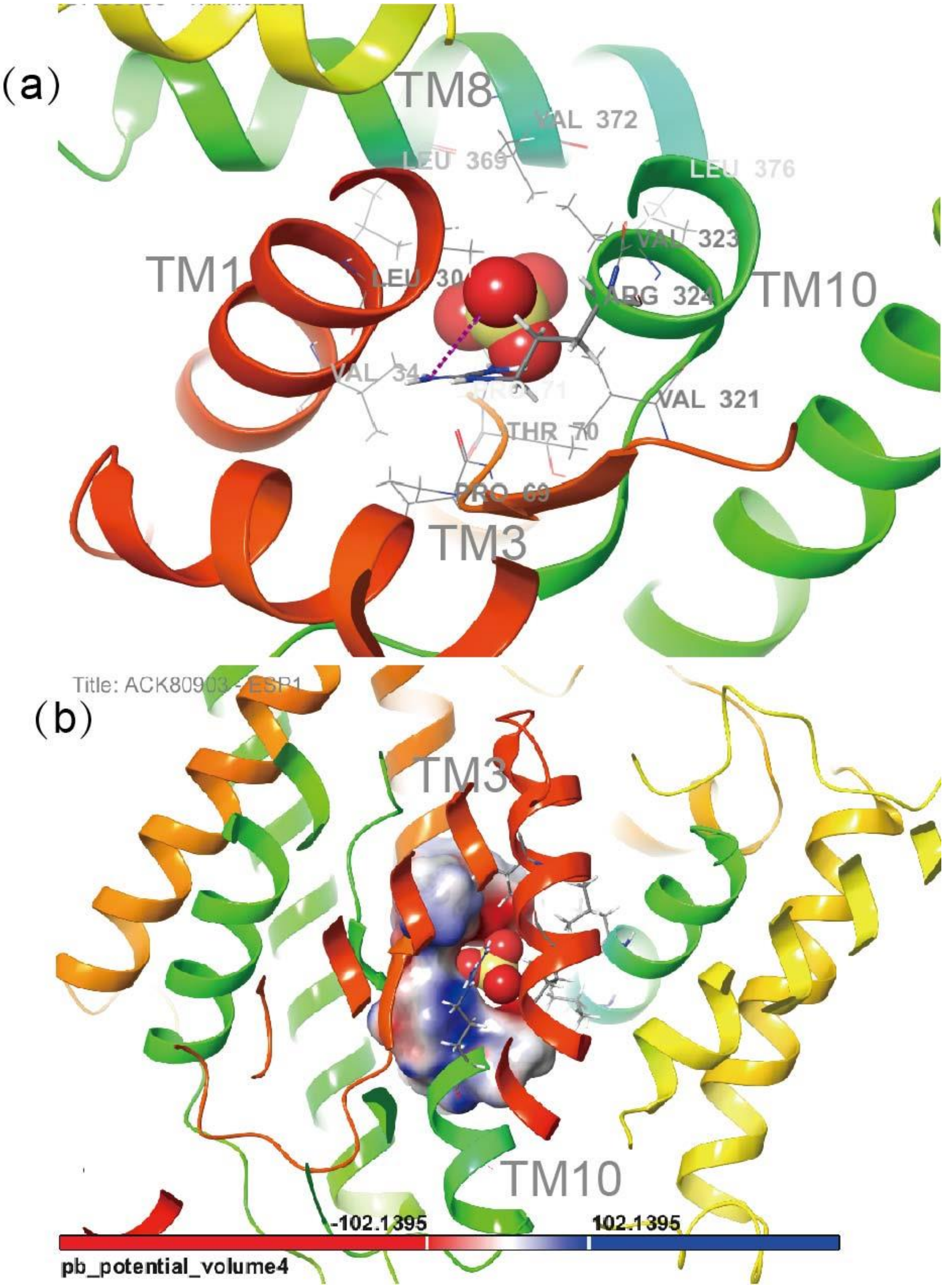

Fig. S5: Structure cut-open view of putative key residues in ACK80903 sulfate transporter (AfSULTR) of *Acidithiobacillia*: (a) The sulfate ion locates in the AtSULTR binding pocket and putatively interacts with surrounding residues (direct interactions are marked with dashed lines); (b) Cut-open view showing the electrostatic potential of the helix dipoles of TM3 and TM10 calculated by adaptive Poisson-Boltzmann solver (APBS), which carry the positive electrostatic potential ends that binds the oxygen anions of sulfate ion;

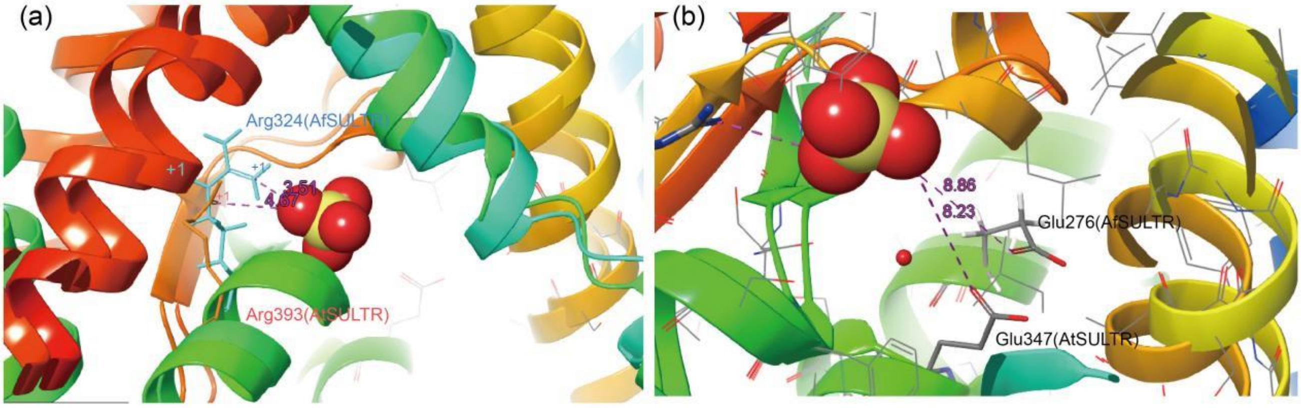

Fig. S6: Structure cut-open view of putative key residues in ACK80903 sulfate transporter (AfSULTR) of *Acidithiobacillia* in comparison to the chloroplastic sulfate transporter of *Arabidopsis thaliana* (AtSULTR, PDB: 7LHV): (a) a conserved Arg324 (Arg393 of AtSULTR) with positive electrostatic potential that forms a putative salt bridge with the bound sulfate ($SO4^{2-}$); (b) a Glu276 in AfSULTR approximately at the same position of AtSULTR Glu347 significant for anion transport and H^+ gradient sensing.

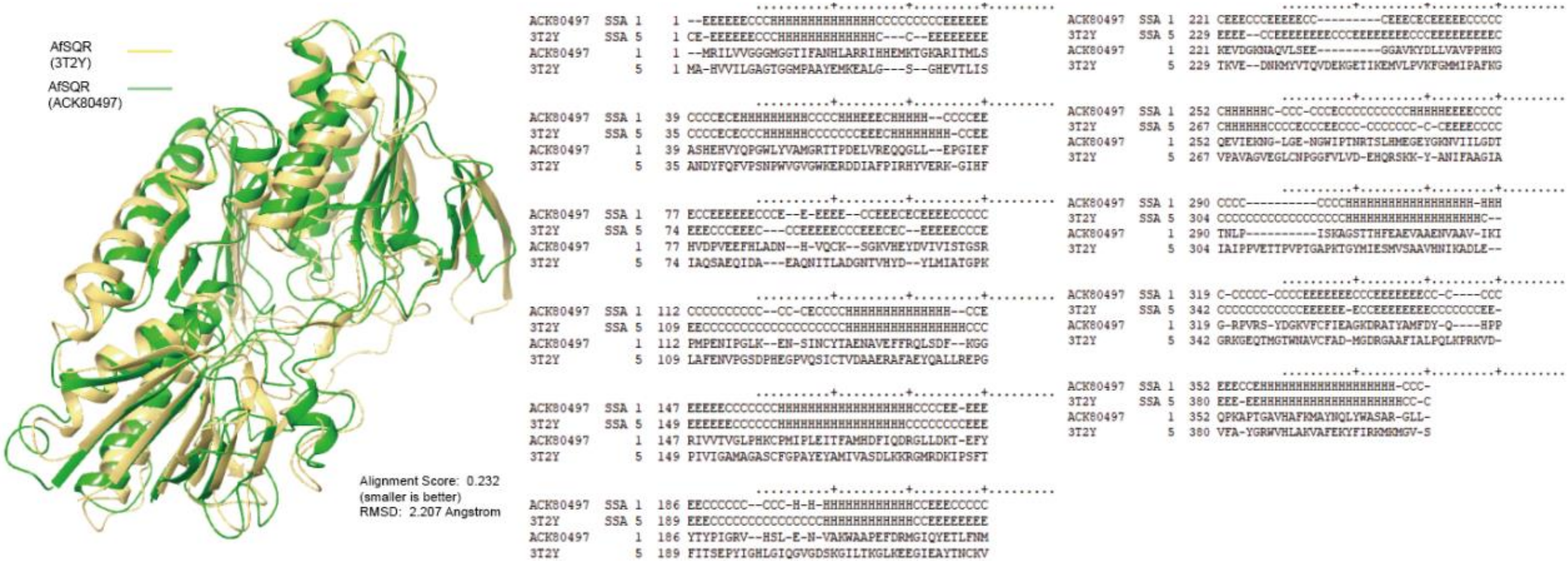

Fig. S7: Structure and sequence alignment of ACK80903 sulfate transporter (AfSULTR) of *Acidithiobacillia* monomer and the chloroplastic sulfate transporter of *Arabidopsis thaliana* (AtSULTR, PDB: 7LHV) monomer. Secondary structure abbreviation: helix, sheet, and coil are abbreviated as H, E, and C.

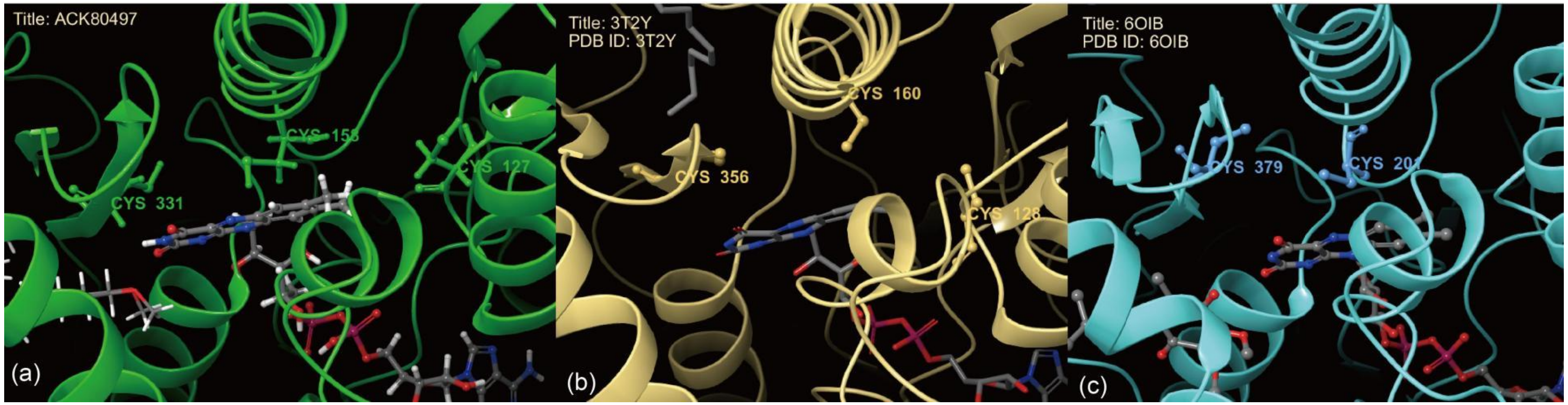

Fig. S8: Comparisons of conserved triad active residues in SQR: (a) the conserved triad active residues of AfSQR-ACK80497, Cys127, Cys158 and Cys331 that locate on a side of the cofactor FAD; (b) Cys128, Cys160 and Cys356 of AfSQR-3T2Y and (c) Cys201, Cys379 of human SQR-6OIB.

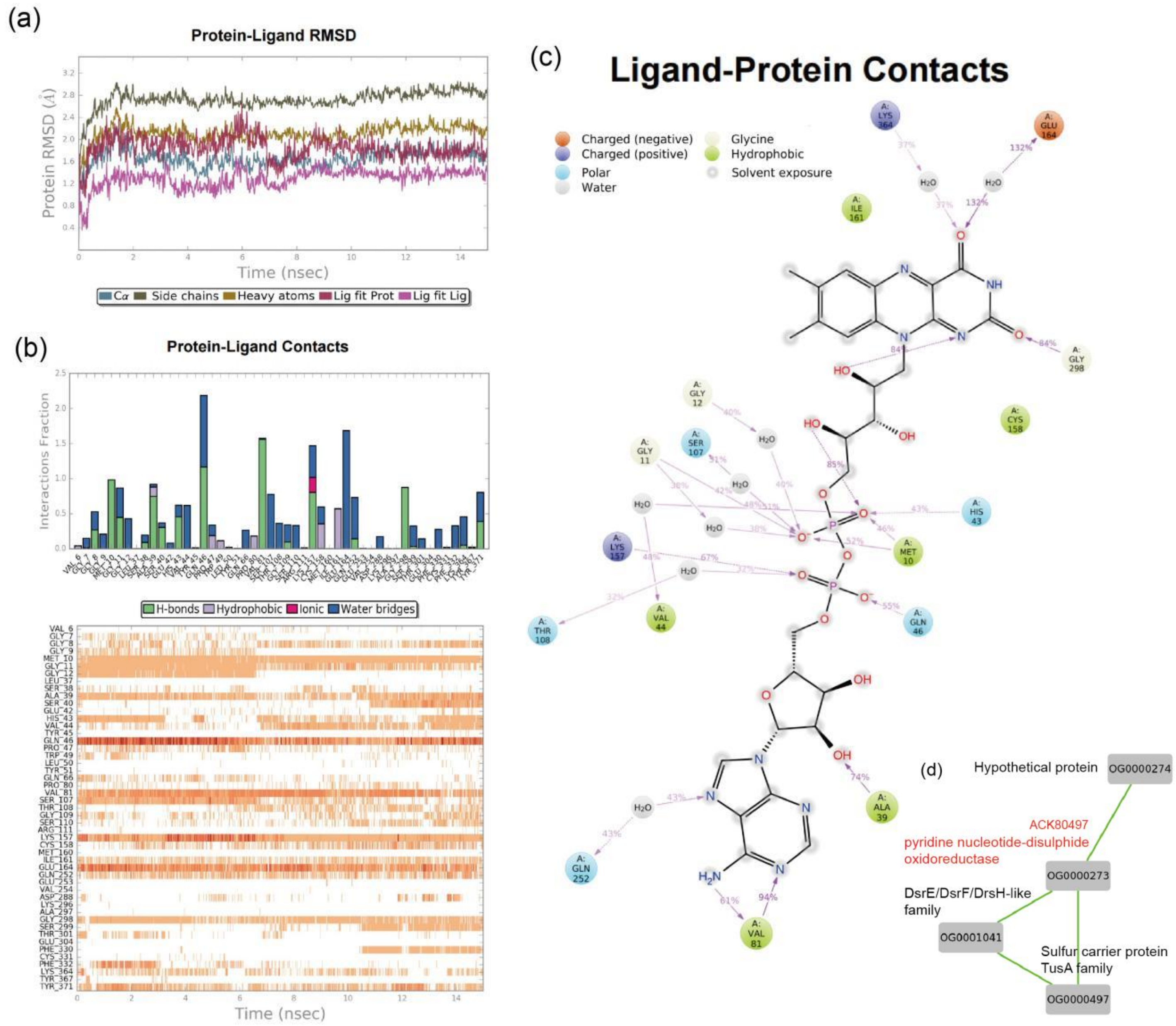

Fig. S9: Classical molecular dynamics (MD) simulation (15 ns) of AfSQR-ACK80497 applied to analyze the putative ligand-protein contacts: (a) The Root Mean Square Deviation (RMSD) during 15ns MD simulation, which shows that the system reach equilibrium at around 2ns. RMSD measures the average change in displacement of a selection of atoms for a particular frame with respect to a reference frame. (b) Protein interactions with the ligand monitored throughout the simulation. These interactions can be categorized by type and summarized, as shown in the legend (top panel). Protein-ligand interactions (or 'contacts') are categorized into four types: Hydrogen Bonds, Hydrophobic, Ionic and Water Bridges. The stacked bar charts are normalized over the course of the trajectory. The bottom panel shows a timeline representation of the interactions and contacts and which residues interact with the ligand in each trajectory frame. Some residues make more than one specific contact with the ligand, which is represented by a darker shade of orange. (c) A schematic of detailed ligand atom interactions with the protein residues. Interactions that occur more than 30.0% of the simulation time in the selected trajectory (0.00 through 15.00 ns), are shown. (d) The gene

co-occurrence sub-network showing the neighbors of protein ACK80497 (spearman *cor* >0.7, *p*<0.05).

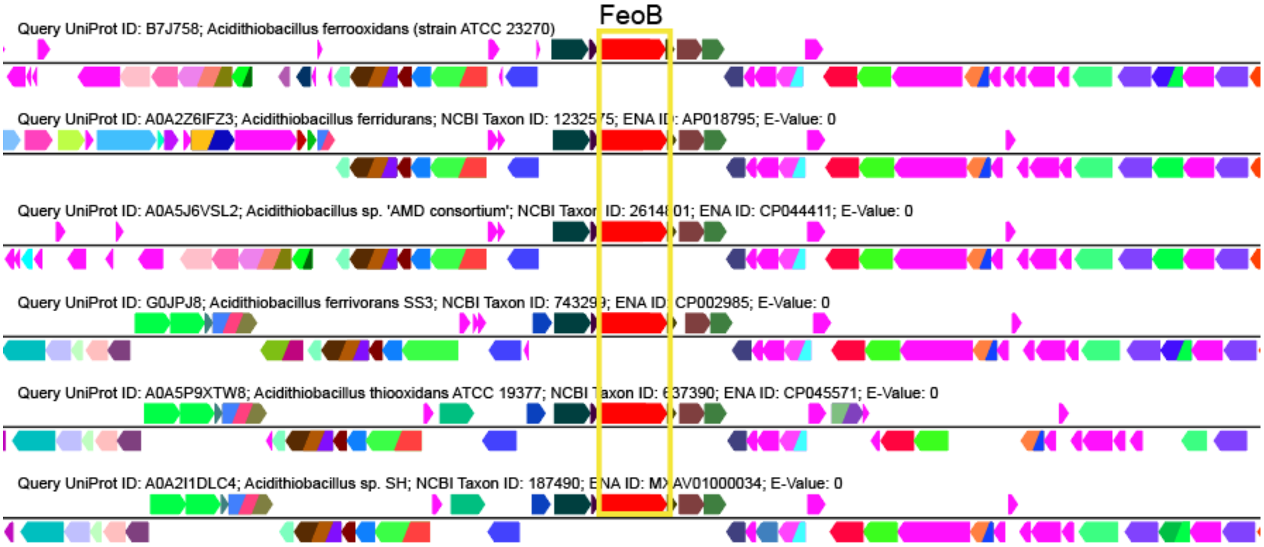

Fig. S10: Genome context comparisons of ACK79582 ferrous iron transporter (AfFeoB) in *Acidithiobacillia*, which suggest a conserved evolutionary relation.

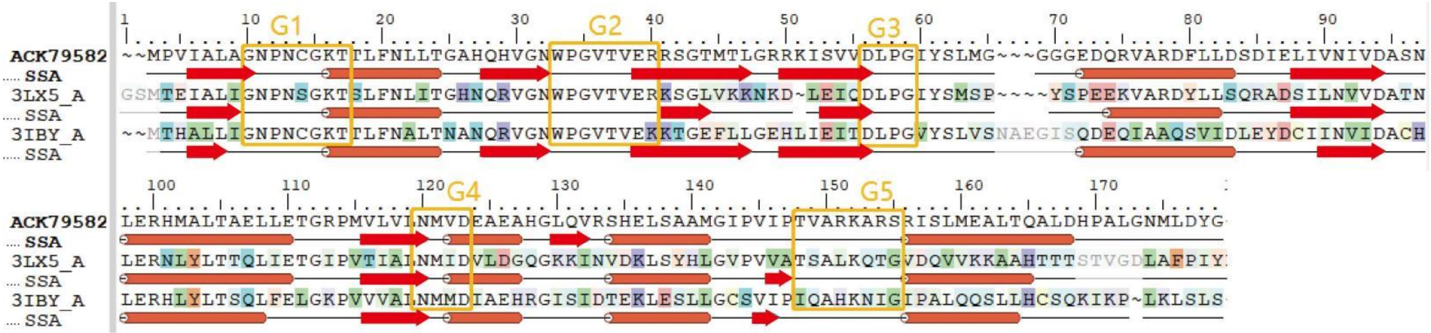

Fig. S11: Multiple sequence alignment of GTPase motifs G1-G5 (marked with yellow rectangles) from AfFeoB (ACK79582), *Streptococcus thermophilus* NFeoB (PDB: 3LX5), and *Legionella pneumophila* FeoB (PDB: 3IBY)

Title: AfFeoB (ACK79582)

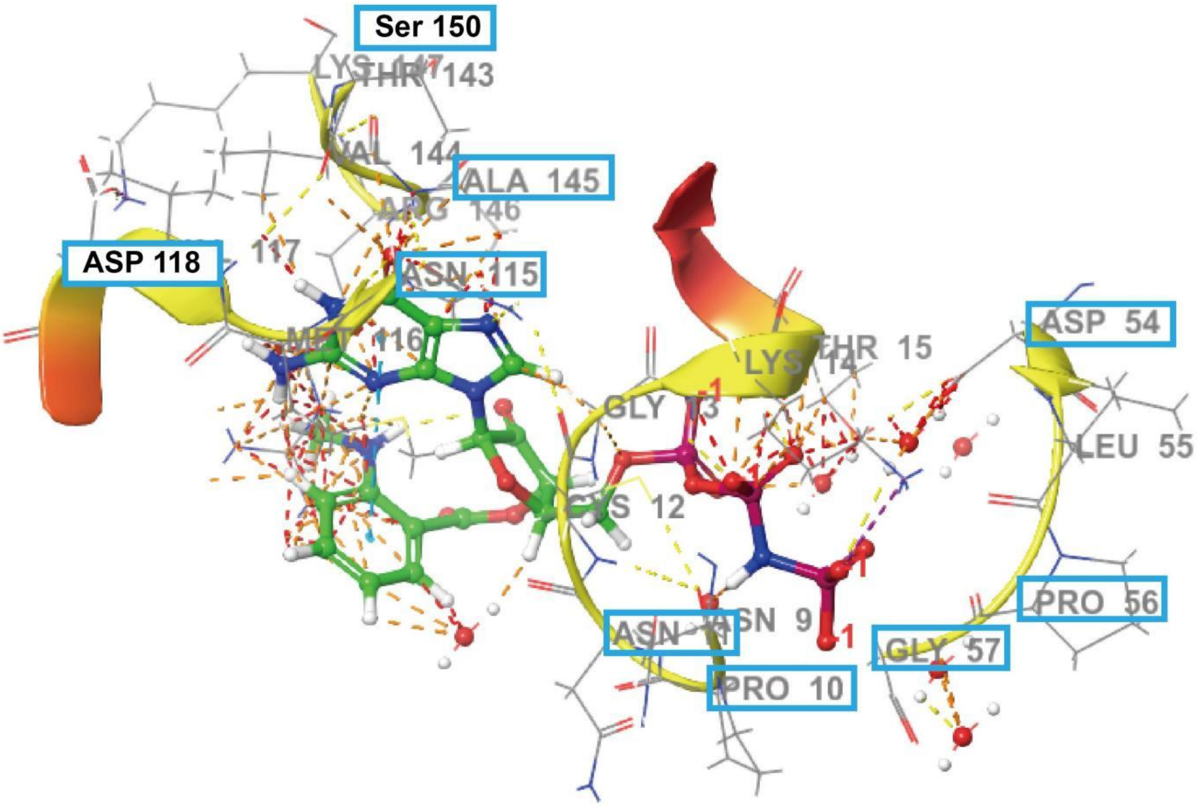

PDB ID: 3LX5

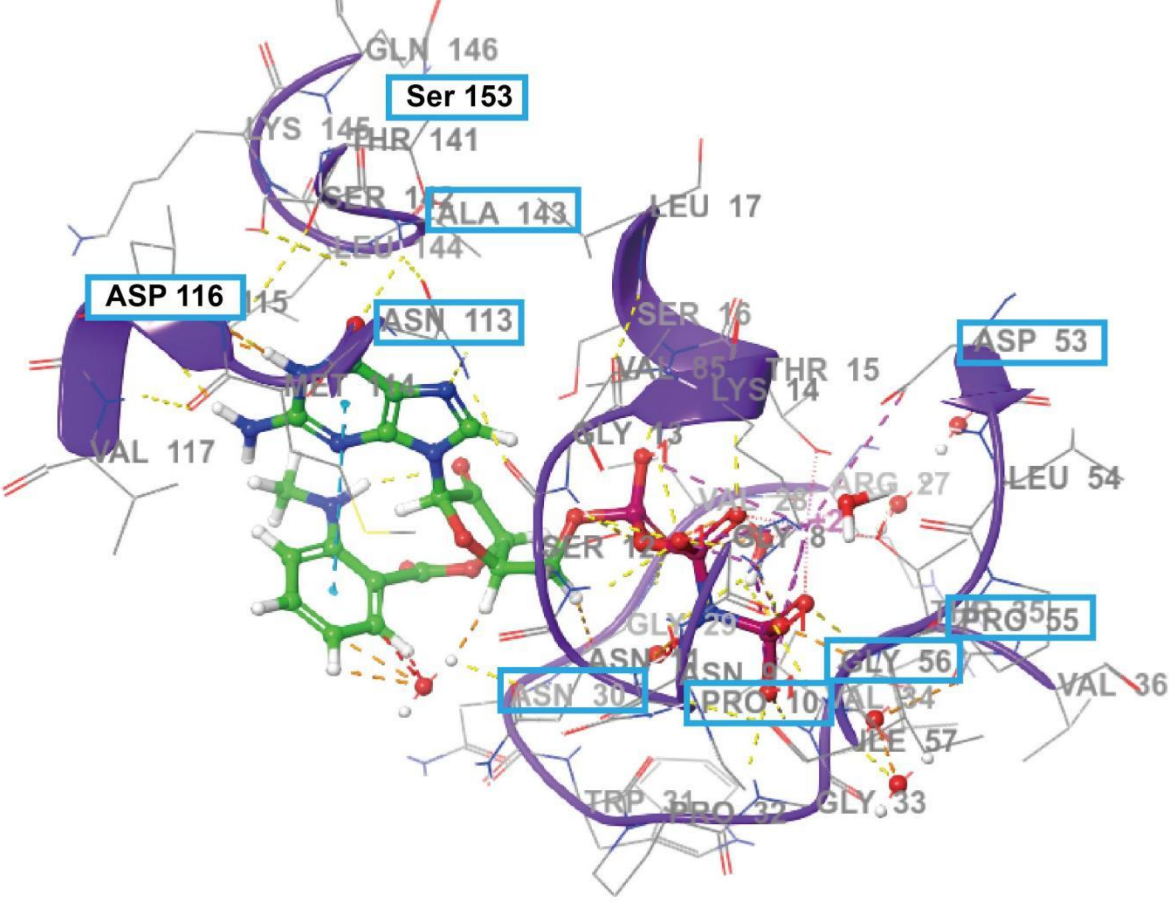

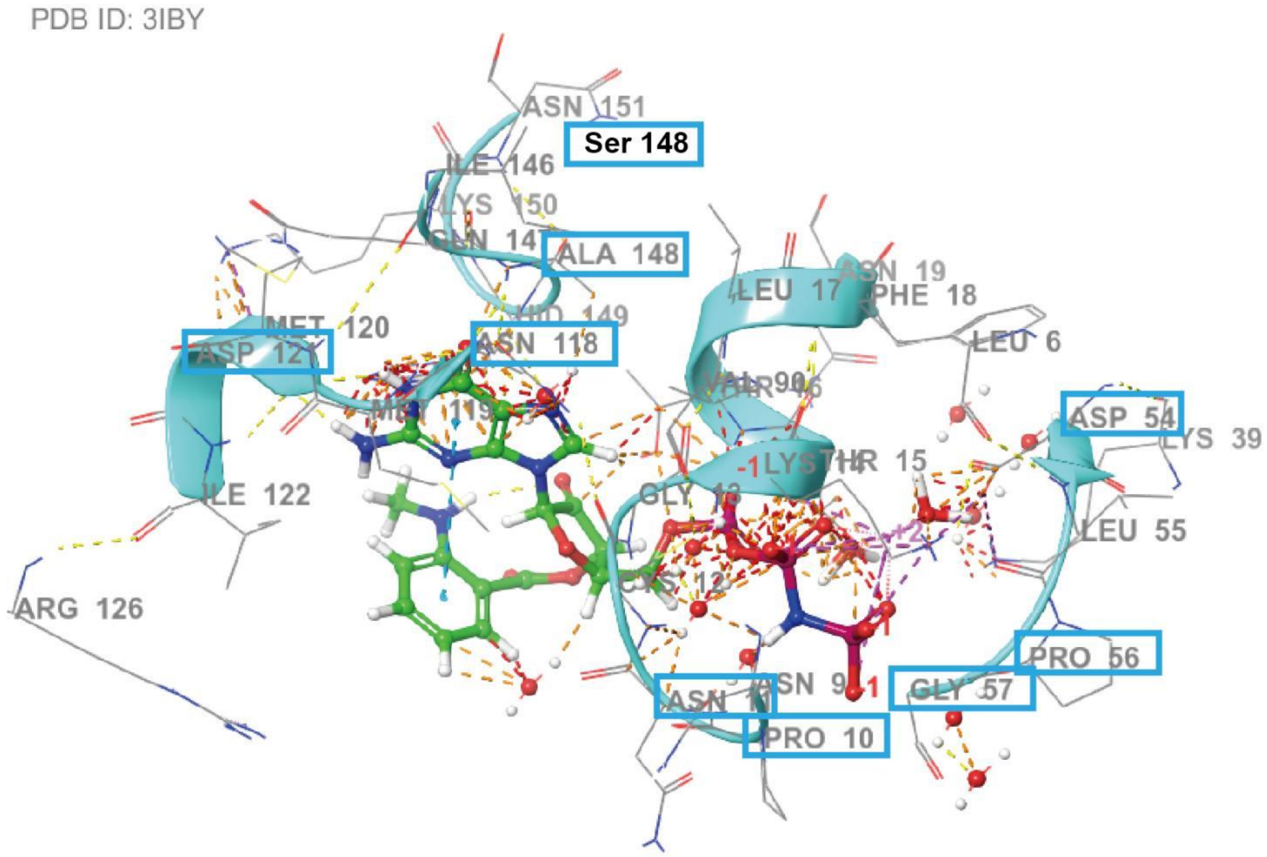

Fig. S12: Comparisons of the residues surrounding the nucleotide-binding site of FeoB structures of *Acidithiobacillia* (ACK79582, top) *Streptococcus thermophilus* LMG 18311 (PDB 3LX5, middle) and *Legionella pneumophila* (PDB 3IBY, bottom). The GDP molecule is shown in ball and stick shape., Key conserved residues are highlighted with blue rectangles

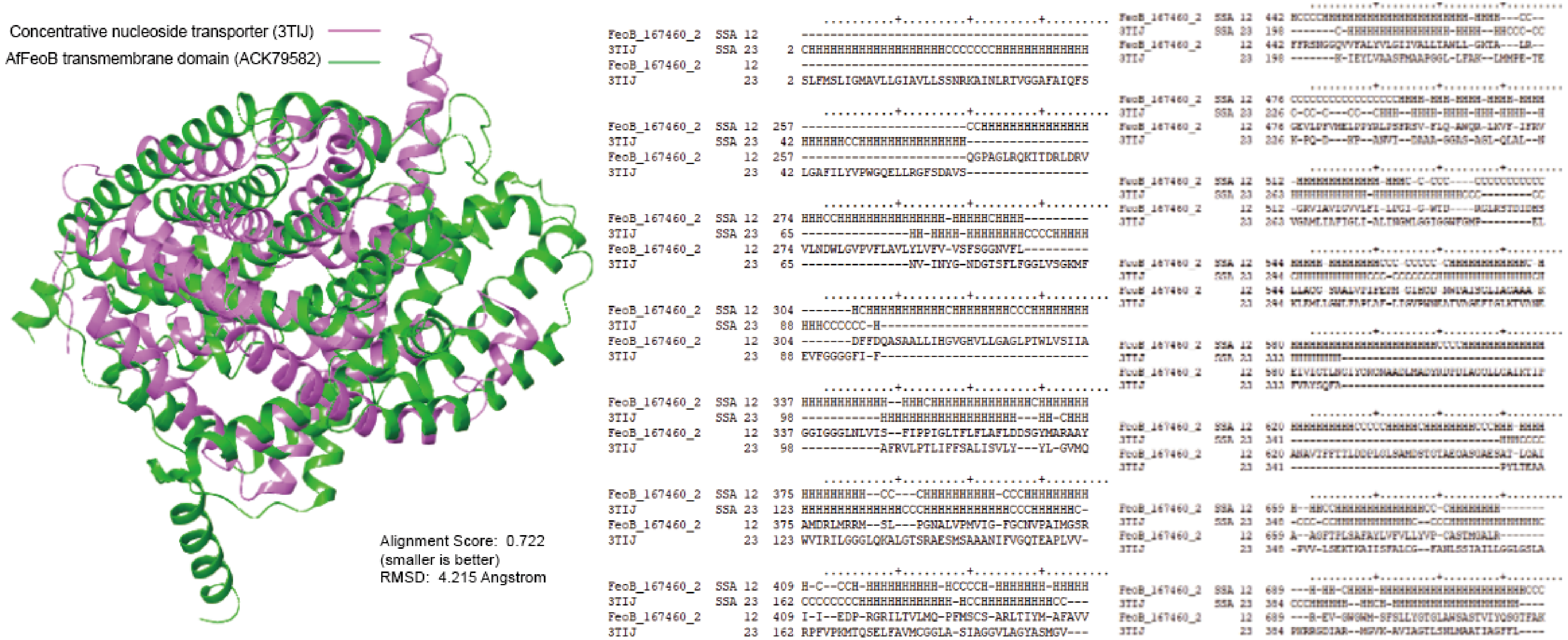

Fig. S13: Structure and sequence alignment of the transmembrane domain of ACK79582, a ferrous iron transport membrane protein FeoB (AfFeoB) of *Acidithiobacillia* to the reported concentrative nucleoside transporter vcCNT (PDB 3TIJ). Secondary structure abbreviation: helix, sheet, and coil are abbreviated as H, E, and C.

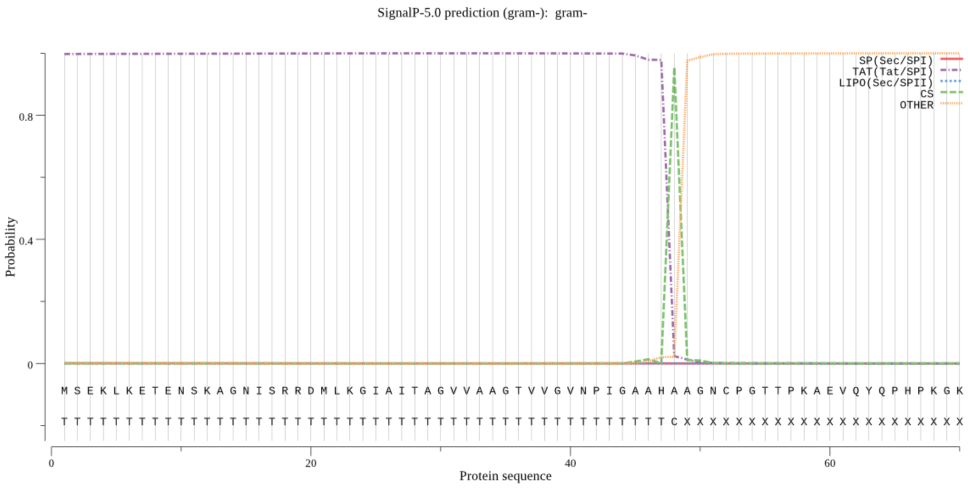

Fig. S14: SignalP v.5.0 signal peptide prediction of the iron oxidase (Iro, accession: ACK79288) in *Acidithiobacillia*, which shows that: the type of signal peptide is TAT(TAT /SPI) with a existence probability of 95.5%; the cutting site is between residues 48-49; the predicted signal peptide sequence is MSEKLKETENSKAGNISRRDMLKGIAITAGVVAAGTVVGVNPIGAAHA.

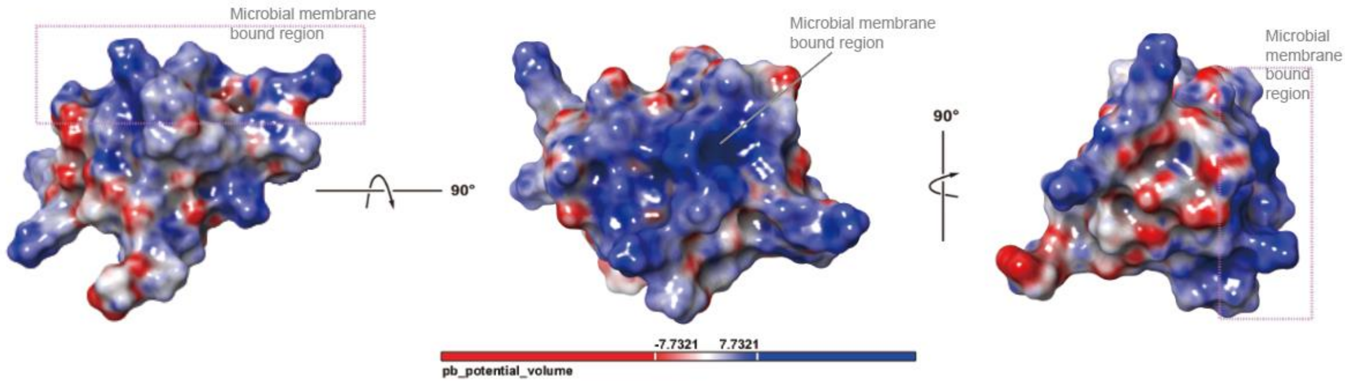

Fig. S15: Electrostatic potential surfaces of the overall iron oxidase monomer (Iro, Genbank: ACK79288) in *Acidithiobacillia* calculated with adaptive Poisson-Boltzmann solver (APBS), which is rotated 90° downward and rightward as indicated to reveal location of the negatively charged enriched region that putatively bind to membrane.

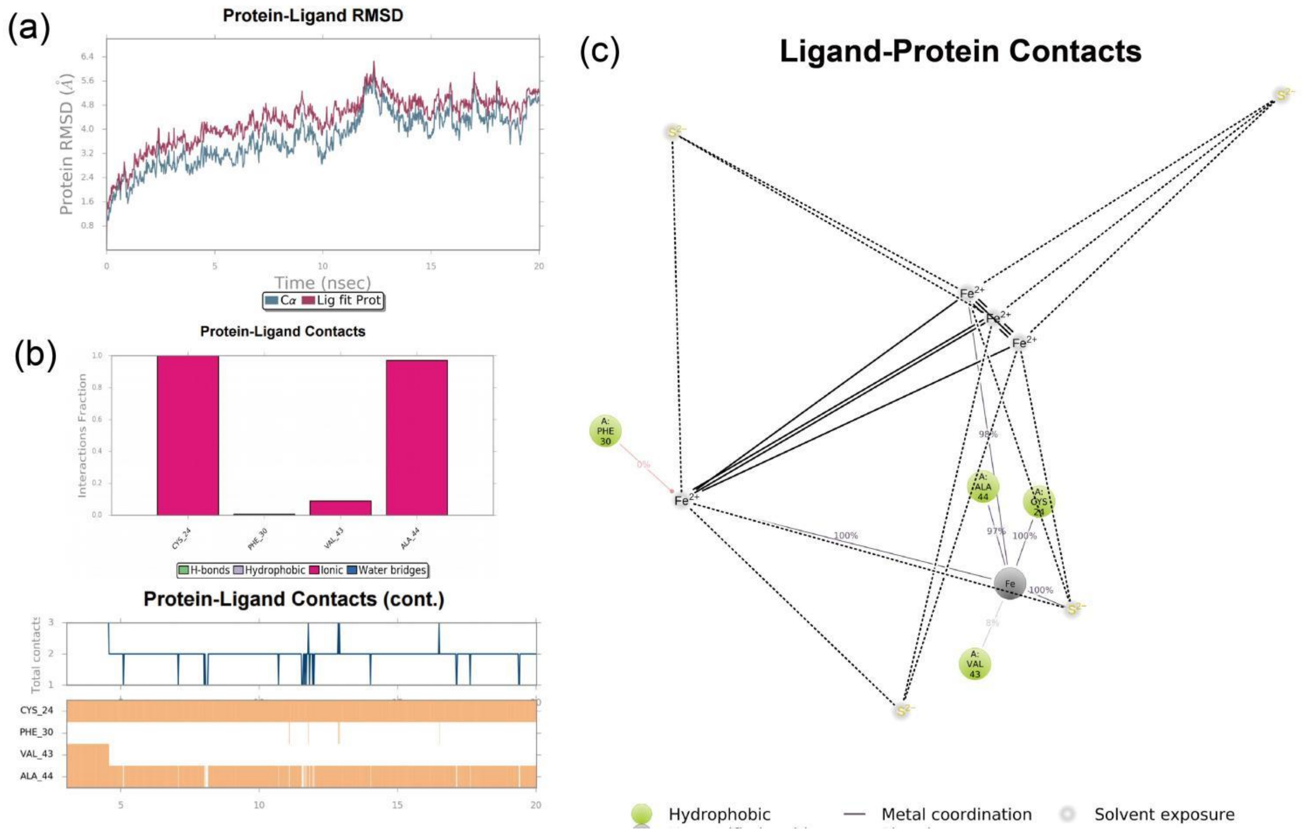

Fig. S16: Classical molecular dynamics (MD) simulation (20 ns) of iron oxidase (Iro, ACK79288) applied to analyze the putative ligand-protein contacts: (a) The Root Mean Square Deviation (RMSD) during 20 ns MD simulation, which shows that the system reach equilibrium at around 2ns. RMSD measures the average change in displacement of a selection of atoms for a particular frame with respect to a reference frame. (b) Protein interactions with the ligand monitored throughout the simulation. These interactions can be categorized by type and summarized, as shown in the legend (top panel). The bottom panel shows a timeline representation of the interactions and contacts and which residues interact with the ligand in each trajectory frame. Some residues make more than one specific contact with the ligand, which is represented by a darker shade of orange. (c) A schematic of detailed ligand atom interactions with the protein residues. Interactions that occur more than 30.0% of the simulation time in the selected trajectory (0.00 through 20.00 ns), are shown.

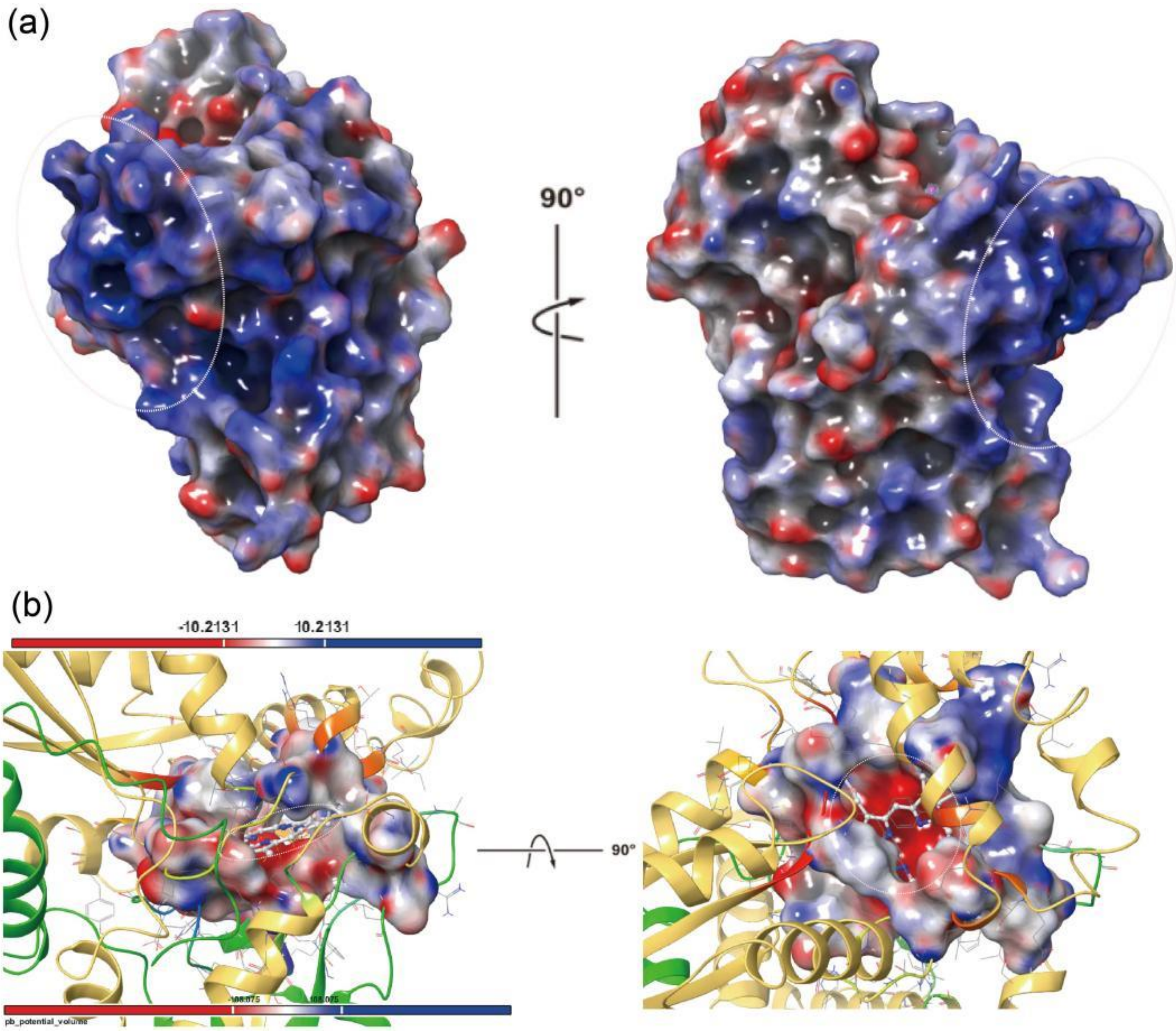

Fig. S17: Electrostatic potential surfaces of ferrochelatase in *A. ferrooxidans* ATCC23270: (a) Electrostatic potential surfaces of the overall monomer of ferrochelatase (genbank ACK80603) in *Acidithiobacillia* calculated with adaptive Poisson-Boltzmann solver (APBS), which is rotated 90° rightward as indicated to reveal location of the positively charged enriched region that putatively bind to ferrous iron. (b) The negatively charged region in the protoporphyrin binding pocket face of ferrochelatase in *Acidithiobacillia* (genbank ACK80603) calculated with adaptive Poisson-Boltzmann solver (APBS), which is rotated 90° downward for a comprehensive display.

(a)

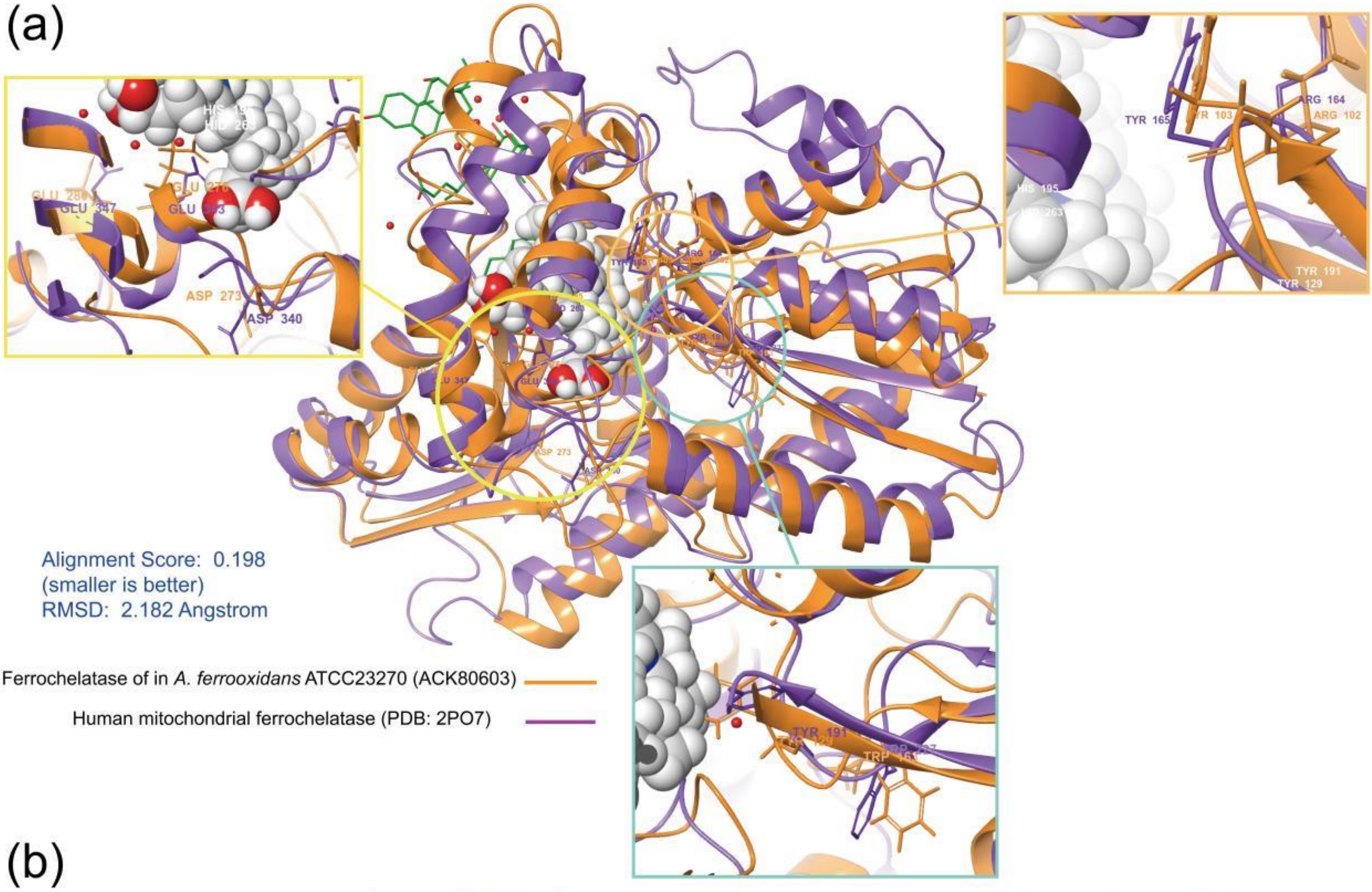

(b)

```
                                  ..........+.........+.........+.........
ACK806031  SSA 15     1 --E-EEEEEEECCCCCCCHHHHHHHHHHHHHHCCCCCCC
2PO7       SSA 16    65 CCCEEEEEEEEECCCCCHHH--HHCHHHHHHHHHHCCCC--C
ACK806031      15     1 --M-IGILVVNLGTPDAPTAPAVRRYLREFLSDTRVVELP
2PO7           16    65 RKPKTGILMLNMGGPETLG--DVHDFLLRLFLDQDLM--T

                                  ..........+.........+.........+.........
ACK806031  SSA 15    38 HHH-HHHHHHHHCCCHHHHHHHHHHHHHHCCCC-CCHHHHH
2PO7       SSA 16   101 C-CCHHHHHHH-HHHHHHHCHHHHHHHH-C--CCCCHHHH
ACK806031      15    38 RML-WWPILNGPILRFRPARSARNYASIWLPD-GSPLMVY
2PO7           16   101 L-PIQNKLAPF-IAKRLTPKIQEQYRRI-G--GGSPIKIW

                                  ..........+.........+.........+.........
ACK806031  SSA 15    76 HHHHHHHHHHHHHHHCCC--EEEEEEEEECCCCCHHHHHH
2PO7       SSA 16   136 HHHHHHHHHHHHHHHHCCCCCEEEEEEEEECCCCCCHHHHHH
ACK806031      15    76 SQRQCDALQARWDRQFPG--QVRVKLAMRYGNPSVAKGMA
2PO7           16   136 TSKQGEGMVKLLDELSPNTAPHKYYIGFRYVHPLTEEAIE

                                  ..........+.........+.........+.........
ACK806031  SSA 15   114 HHHHHCCEEEEEEEEECCCCCCCHHHHHHHHHHHHHHHH-C
2PO7       SSA 16   176 HHHHHCCEEEEEEEEECCCCCHHHHHCHHHHHHHHHHHHHCC
ACK806031      15   114 ALRDAGCGRILVVPLYPQYAAATTASTFDAVAKELRKW-R
2PO7           16   176 EMERDGLERAIAFTQYPQYSCSTTGSSLNAIYRYYNQVGR

                                  ..........+.........+.........+.........
ACK806031  SSA 15   153 CC-CEEECCCCCCCCHHHHHHHHHHHHHHHHHHH-CC-C--
2PO7       SSA 16   216 CCCCEEEEECCCCCCHHHHHHHHHHHHHHHHH-HHHCCCCC
ACK806031      15   153 EI-PEMRMIRDWHAHPAYIGALADSIRAWWQAH-GQ-A--
2PO7           16   216 KPTMKWSTIDRWPTHHLLIQCFADHILKELD-HFPLEKRS

                                  ..........+.........+.........+.........
ACK806031  SSA 15   188 C-EEEEEEEECCHHHHHHCCHHHHHHHHHHHHHHHHHHC--
2PO7       SSA 16   255 CCEEEEEEEEEECHHHHHHCCHHHHHHHHHHHHHHHHHHHCCC
ACK806031      15   188 E-RLLITFHGLPESSVLKGDPYRAQCEKTTALLVQALG--
2PO7           16   255 EVVILFSAHSLPMSVVNRGDPYPQEVSATVQKVMERLEYC

                                  ..........+.........+.........+.........
ACK806031  SSA 15   225 CCCCEEEEEEEECCCCCCCCCCCCHHHHHHHHHHHCCEEEE
2PO7       SSA 16   295 CE---EEEEEECCCCCCCCECCCHHHHHHHHHHHHCCEEEE
ACK806031      15   225 LADDQWVQSFQSRFGAARWLEPYTDRTLVGLARAGVSRVD
2PO7           16   295 NP---YRLVWQSKVGPMPWLGPQTDESIKGLCERGRKNIL

                                  ..........+.........+.........+.........
ACK806031  SSA 15   265 EECCCCCCCCHHHHHHHHHHHC-HHHHHHCCCEEECCCCC
2PO7       SSA 16   332 EECCCCCCCHHHHHHHHHCHHHHHHHCCCCCCCEEECCCCC
ACK806031      15   265 AVCPGFAADCVETLQEIALEGK-ETFLHAGGQELRYIPAL
2PO7           16   332 LVPIAFTSDCIETLYELDIEYSQVLAKECGVENIRRAESL

                                  ..........+.........+.........+.........
ACK806031  SSA 15   304 CCCHHHHHHHHHHHHHHHHHHCCCC-CCCCCCCCCC-----
2PO7       SSA 16   372 CCCHHHHHHHHHHHHHHHHHHH-CCCCCCCCC-----CCCCC
ACK806031      15   304 NDNPLWIAAFSRILEPHWRDWDAL-ENFAVSPESG-----
2PO7           16   372 NGNPLFSKALADLVHSHIQS-NELCSKQLT-----LSCPL

                                  ..........+.........+.........+.........
ACK806031  SSA 15       ------------------
2PO7       SSA 16   406 CCCHHHHHHHHHHHHHHCC
ACK806031      15       ------------------
2PO7           16   406 CVNPVCRETKSFFTSQQL
```

Fig. S18: (a) Comparisons of overall structure and key residues putative involved in enzymatic reaction between ferrochelatase in *Acidithiobacillia* (ACK80603) and human mitochondrial ferrochelatase (PDB: 2PO7); The substrate protoporphyrin molecule is shown in ball shape; (b) Sequence alignment of ACK80603 and the PDB 2PO7. Secondary structure abbreviation: helix, sheet, and coil are abbreviated as H, E, and C.

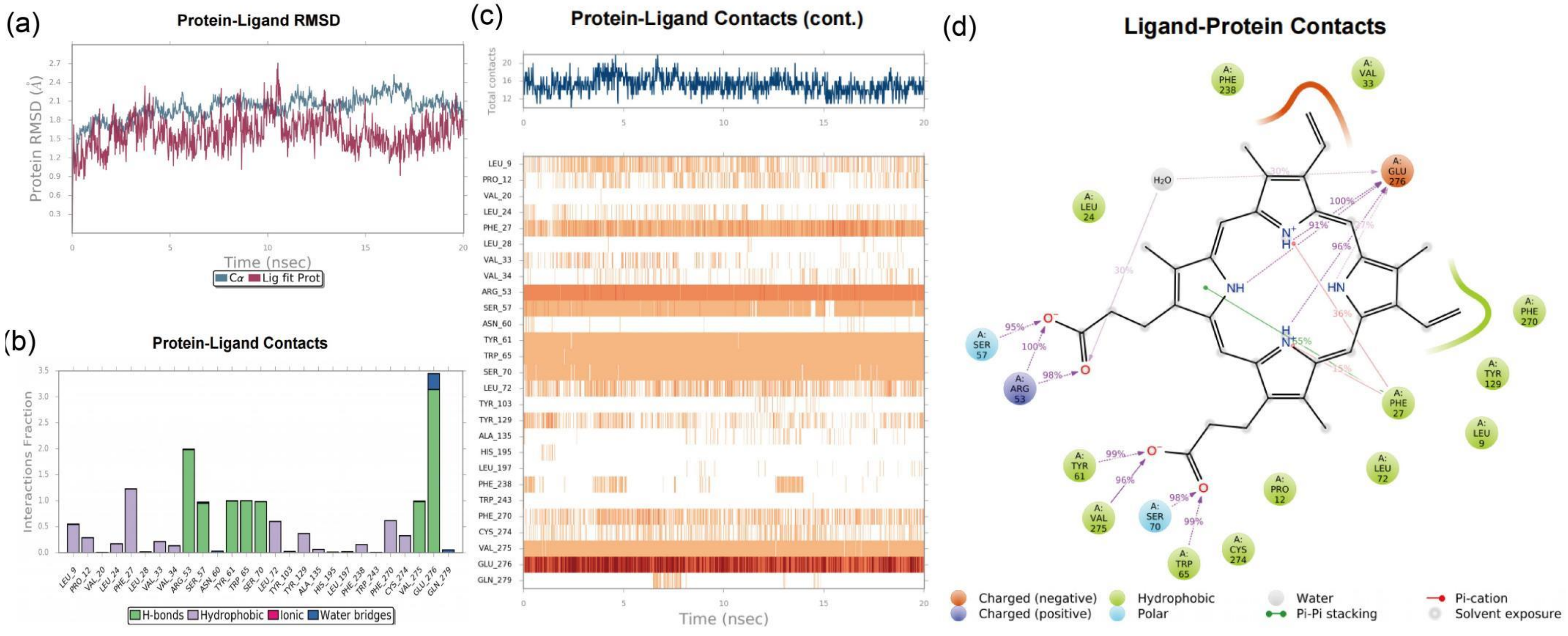

Fig. S19: Molecular dynamics (MD) simulation (20 ns) of ferrochelatase (ACK806038) applied to analyze the putative ligand-protein contacts: (a) The Root Mean Square Deviation (RMSD) during 20 ns MD simulation. RMSD measures the average change in displacement of a selection of atoms for a particular frame with respect to a reference frame. (b) Protein interactions with the ligand monitored throughout the simulation. These interactions can be categorized by type and summarized, as shown in the legend. Protein-ligand interactions (or 'contacts') are categorized into four types: Hydrogen Bonds, Hydrophobic, Ionic and Water Bridges. The stacked bar charts are normalized over the course of the trajectory. (c) Panel shows a timeline representation of the interactions and contacts and which residues interact with the ligand in each trajectory frame. Some residues make more than one specific contact with the ligand, which is represented by a darker shade of orange. (d) A schematic of detailed ligand atom interactions with the protein residues. Interactions that occur more than 30.0% of the simulation time in the selected trajectory (0.00 through 20.00 ns), are shown.

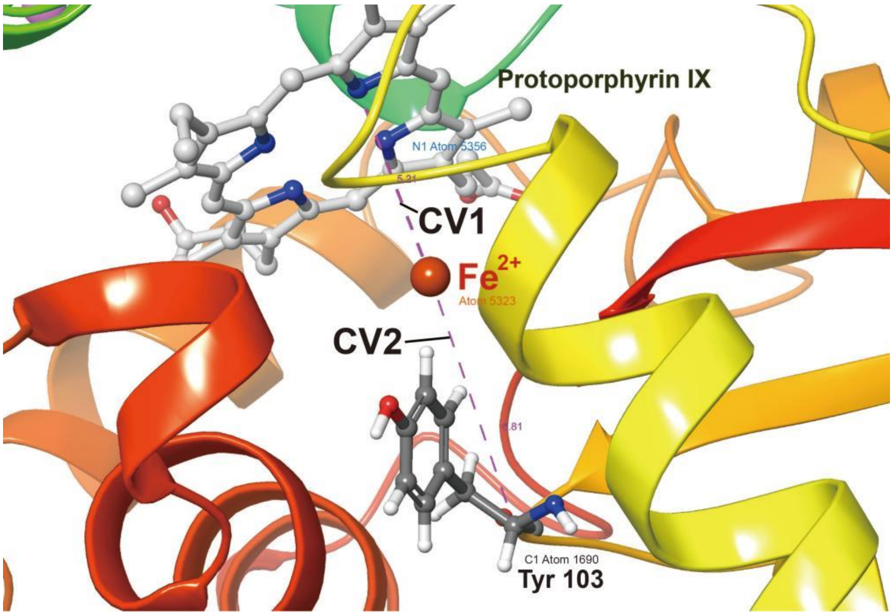

Fig. S20: Atoms chosen as CV for metadynamics simulations in ferrochelatase (ACK806038). The CV1 is the distance between the nitrogen atom (N1) of the protoporphyrin IX molecule and the free ferrous iron ion. The CV2 is the distance between the main chain carbon atom (C1) of the gate residue Tyr103.

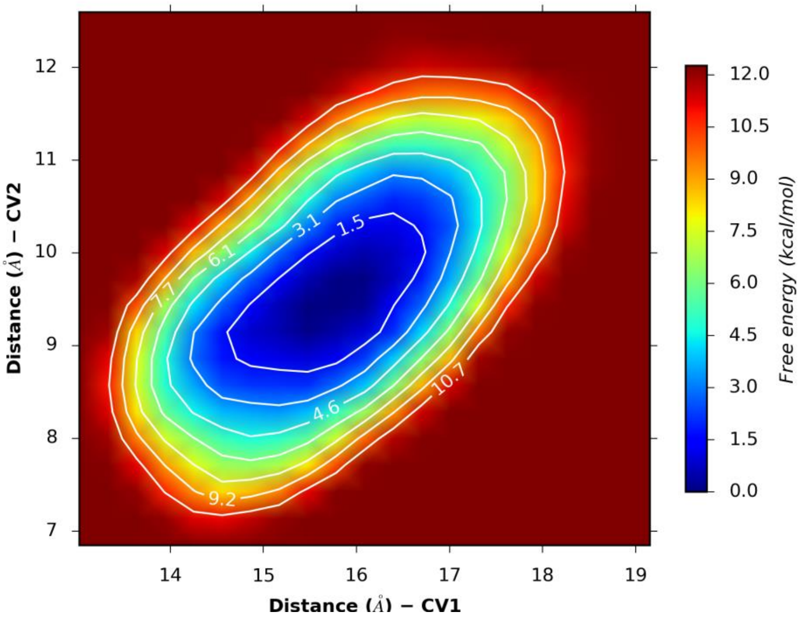

Fig. S21: Two-dimensional free energy profile constructed based on metadynamics (20 ns) of ferrochelatase (ACK806038). The CV1 and CV2 (Fig. S21) are the x- and y-axis, respectively.

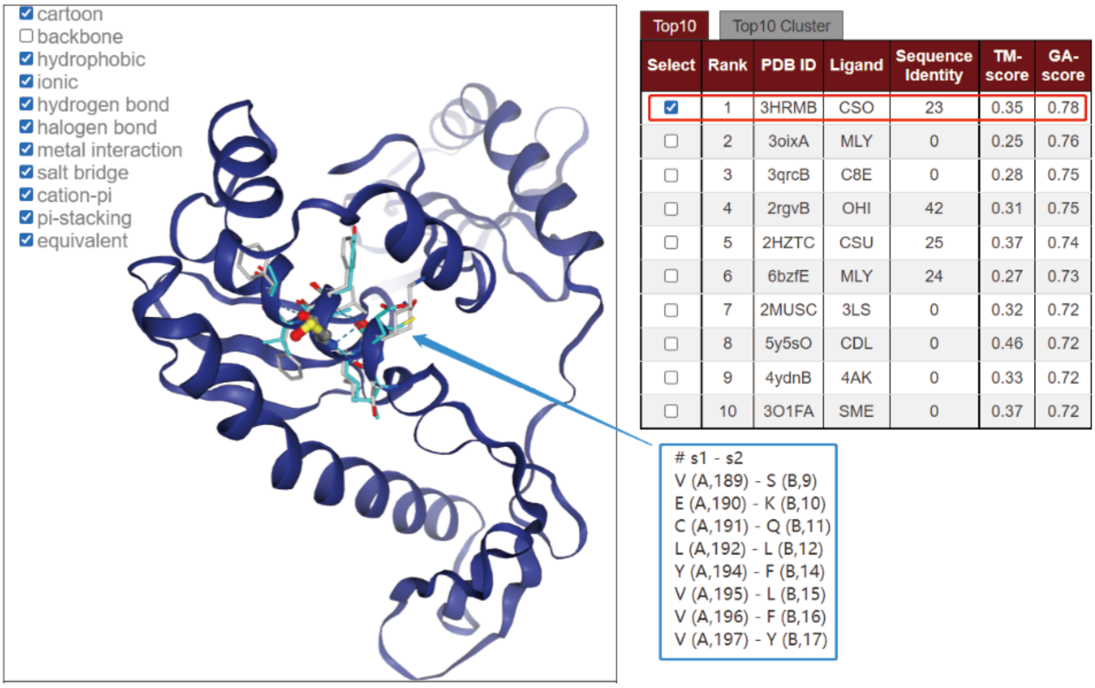

Select	Rank	PDB ID	Ligand	Sequence Identity	TM-score	GA-score
☑	1	3HRMB	CSO	23	0.35	0.78
☐	2	3oixA	MLY	0	0.25	0.76
☐	3	3qrcB	C8E	0	0.28	0.75
☐	4	2rgvB	OHI	42	0.31	0.75
☐	5	2HZTC	CSU	25	0.37	0.74
☐	6	6bzfE	MLY	24	0.27	0.73
☐	7	2MUSC	3LS	0	0.32	0.72
☐	8	5y5sO	CDL	0	0.46	0.72
☐	9	4ydnB	4AK	0	0.33	0.72
☐	10	3O1FA	SME	0	0.37	0.72

Fig. S22: LBS Finder& Refiner result page, which shows predicted substrate binding pocket and selected bound ligand aligned onto the 3D structure of query protein ACK77828 conserved hypothetical protein (left); top 10 aligned templates from the G-LoSA search are displayed with their PDB ID, ligand ID, sequence identity, TM-score, and GA-score (right); Equivalent LBS residues between the query (s1) and template (s2) are also shown (blue rectangle).

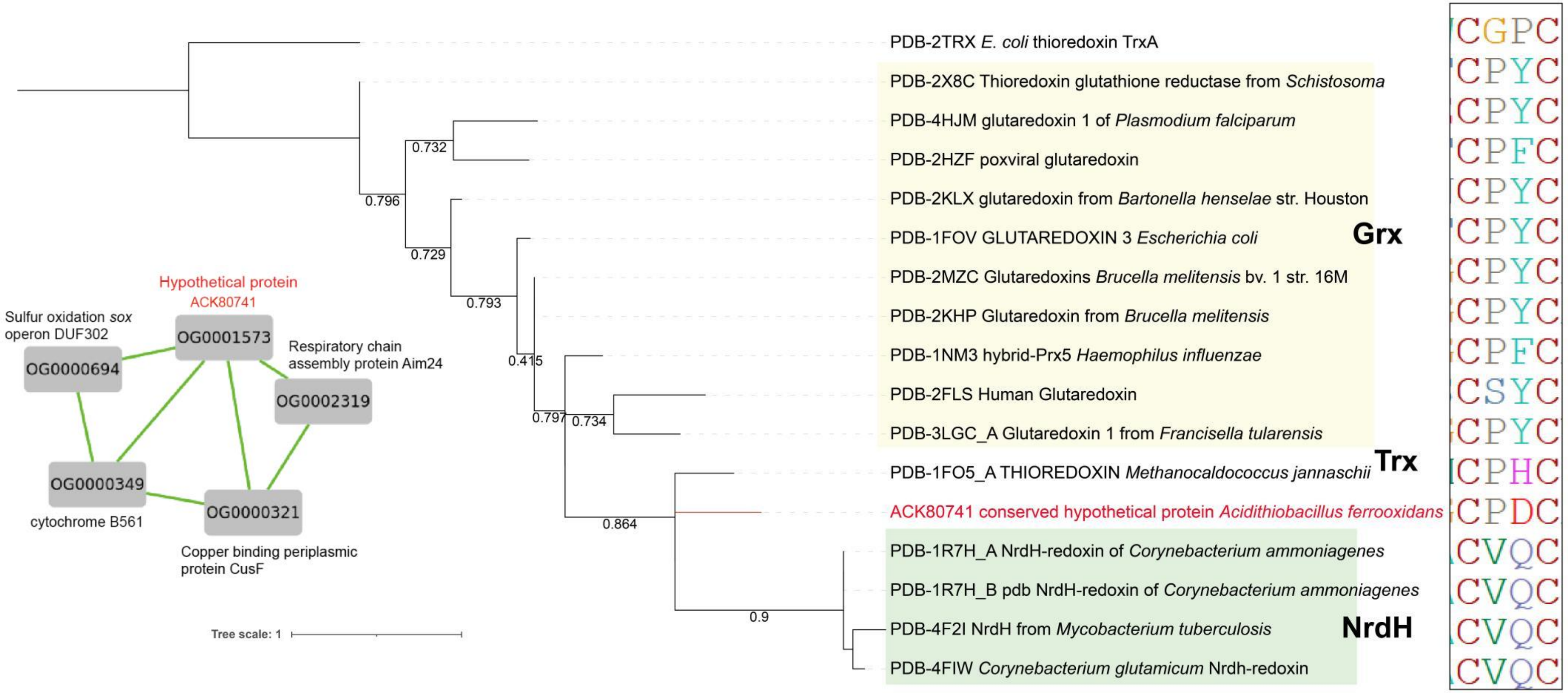

Fig. S23 Maximum likelihood phylogenetic tree (constructed with PhyML program) of protein ACK80741 derived from *Acidithiobacillia* and experimentally confirmed PDB homologs of disulfide oxidoreductase (DSR) family identified with HHsearch and Dali server (http://ekhidna2.biocenter.helsinki.fi/dali/) using ACK80741 as query. Bootstrap values are indicated at each node based on a total of 1,000 bootstrap replicates. Branches representing ACK80741 [*Acidithiobacillia*] are marked in red. The C-X-X-C motifs of the sequences are compared at right panel. The gene co-occurrence sub-network (left) shows the neighbors of protein ACK80741 (spearman *cor* >0.7, p<0.05).

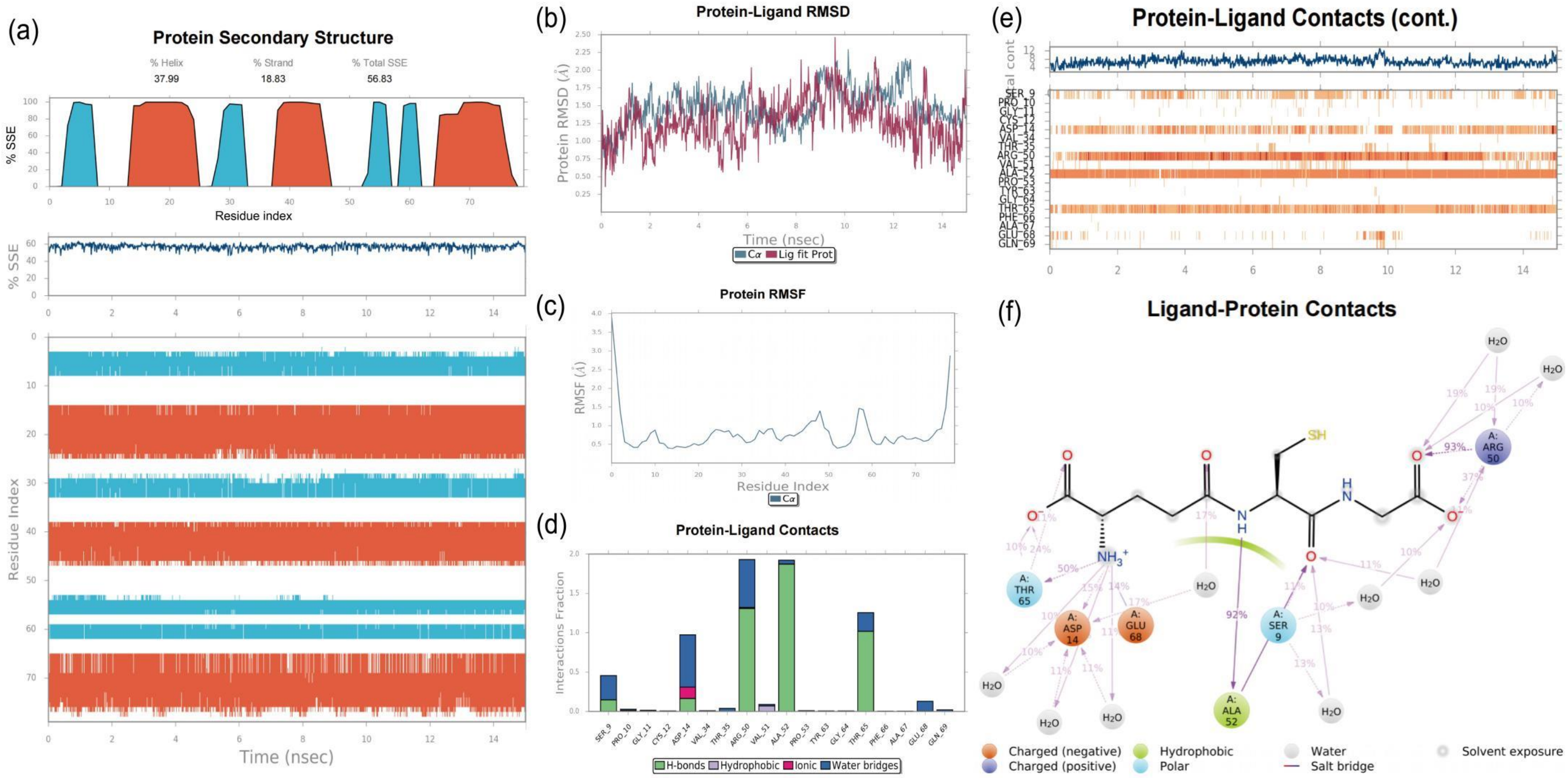

Fig. S24: Molecular dynamics (MD) simulation (15 ns) of ferrochelatase (ACK806038) applied to analyze the putative ligand-protein contacts: (a) Protein secondary structure elements (SSE) like alpha-helices and beta-strands are monitored throughout the simulation. The plot above reports SSE distribution by residue index throughout the protein structure. The plot below summarizes the SSE composition for each trajectory frame over the course of the simulation, and the plot at the bottom monitors each residue and its SSE assignment over time. (b) The Root Mean Square Deviation (RMSD) during 15 ns MD simulation. RMSD measures the average change in displacement of a selection of atoms for a particular frame with respect to the reference frame. (c) The Root Mean Square Fluctuation (RMSF) is useful for characterizing local changes along the protein chain. (d) Protein interactions with the ligand monitored throughout the simulation. The stacked bar charts are normalized over

the course of the trajectory. (e) Panel shows a timeline representation of the interactions and contacts and which residues interact with the ligand in each trajectory frame. Some residues make more than one specific contact with the ligand, which is represented by a darker shade of orange. (f) A schematic of detailed ligand atom interactions with the protein residues. Interactions that occur more than 30.0% of the simulation time in the selected trajectory (0.00 through 15.00 ns), are shown.
